# Synthesis and modelling of the mechanical properties of Ag, Au and Cu nanowires

**DOI:** 10.1080/14686996.2019.1585145

**Published:** 2019-03-22

**Authors:** Nurul Akmal Che Lah, Sonia Trigueros

**Affiliations:** a Innovative Manufacturing, Mechatronics and Sports Lab (iMAMS), Faculty of Manufacturing Engineering, Universiti Malaysia Pahang, Pekan, Malaysia; b Department of Zoology, University of Oxford, Oxford, UK

**Keywords:** Coinage metal nanowires, synthesis method, molecular modelling, nanotechnology, 10 Engineering and Structural materials, 105 Low-Dimension (1D/2D) materials, 106 Metallic materials, 400 Modeling / Simulations, 500 Characterization, Nanomechanical Characterization

## Abstract

The recent interest to nanotechnology aims not only at device miniaturisation, but also at understanding the effects of quantised structure in materials of reduced dimensions, which exhibit different properties from their bulk counterparts. In particular, quantised metal nanowires made of silver, gold or copper have attracted much attention owing to their unique intrinsic and extrinsic length-dependent mechanical properties. Here we review the current state of art and developments in these nanowires from synthesis to mechanical properties, which make them leading contenders for next-generation nanoelectromechanical systems. We also present theories of interatomic interaction in metallic nanowires, as well as challenges in their synthesis and simulation.

## Graphical Abstract

**Figure:**
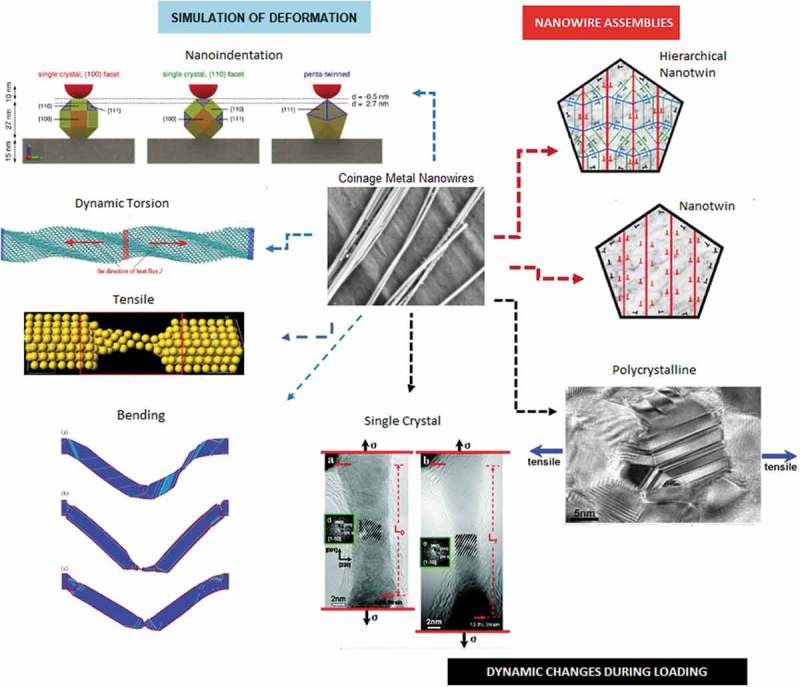

## 1. Introduction

The ability to generate mesoscale metal structures is essential for modern mechanical science and technology since the physical properties of devices are quite diverse and depend on their sub-atomic structure and surface chemistry effects [1–4]. Owing to the continuous miniaturisation of device technologies, 1D metallic nanostructures play a significant role in many applications, acting as basic building blocks for electromechanical systems [1] and also as fillers or nanocontacts to reinforce composite materials in a strident environment [5]. Nevertheless, constructing and determining the mechanical properties of metal nanostructures, which differ from their corresponding bulk ones, remain quite challenging, despite the improvements in characterisation equipment and methods. Therefore, to realise the full potential of 1D metallic nanostructures in numerous applications, for example in field-emitter displays and enantioselective separators, it is essential to identify and resolve the main challenges related to their synthesis, mechanical performance and reliability.

**Figure 1.**
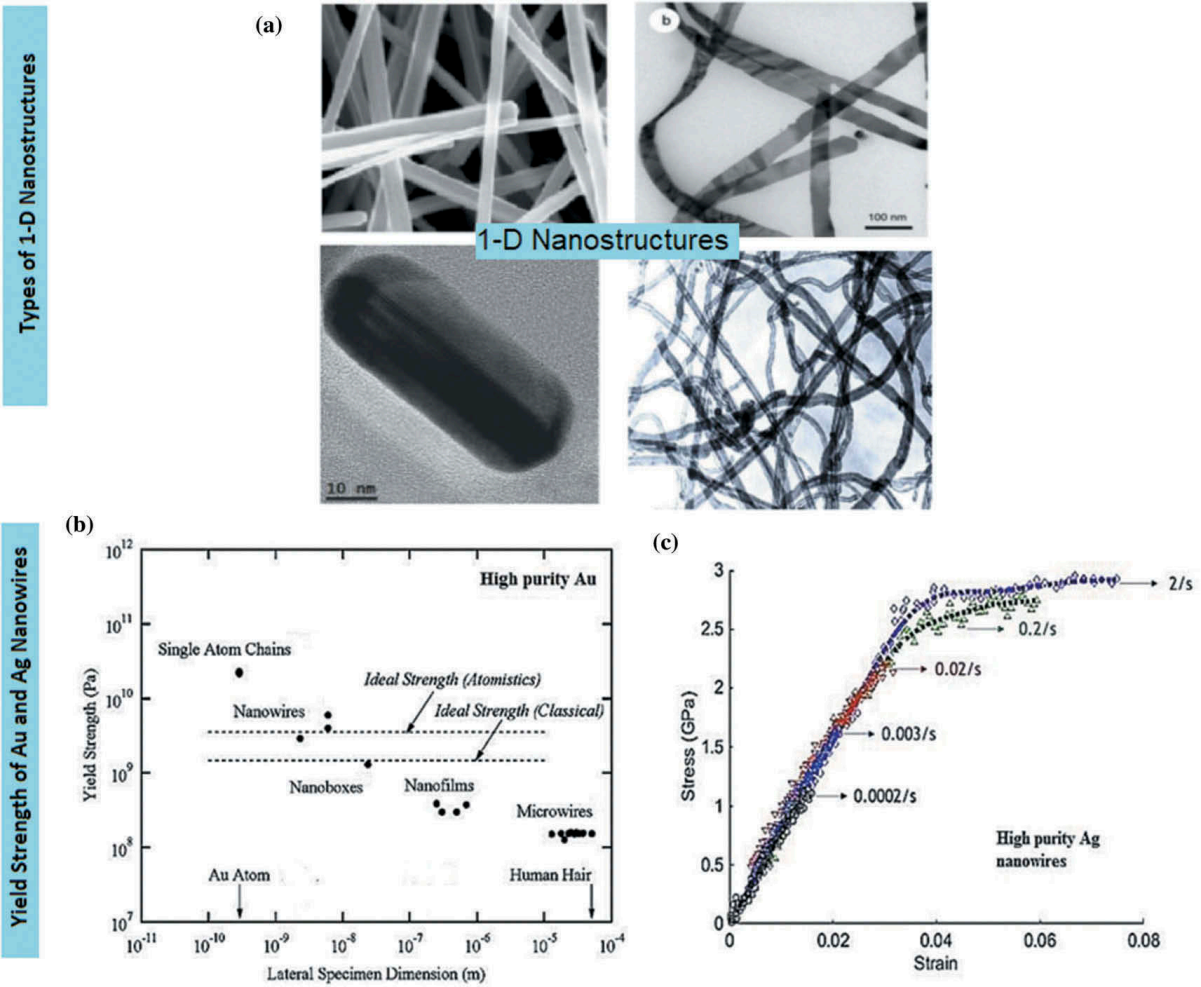
(a) 1D metal nanostructures comprise nanowires, nanobelts, nanorods and nanotubes [26–28]. (b) A collection of experimental measurements and predictions regarding the yield strength of pure Au nanostructures as a function of lateral specimen dimension. Smaller specimens tend to be stronger. At nanoscale length, Au nanowires demonstrate excellent yield strength compared to single-atom chains that typically undergo atomic separation. The relative sizes of Au atom and human hair are included for comparisons [30]. (c) An example of stress vs strain plot of Ag nanowire system at different strain rates [31]. (*Reproduced from* [26] *by permission of the Nature Publishing Group* [27], *by permission of the Royal Society of Chemistry* [28], *Copyright (2013) American Chemical Society* [30], *Copyright (2004) American Chemical Society, reprinted with permission from* [31] *copyright (2016) American Chemical Society*).

Tailored 1D nanostructures, such as ultra-long nanowires, uniform nanorods, nanobelts with diverse facets and nanotubes (Figure 1(a)), are predominantly classified by their physicochemical properties, which are related to morphology. In particular, 1D metallic nanostructures, such as nanowires and nanotubes, have been intensively studied owing to their remarkable nanomechanical characteristics, namely ultra-high specific strength and strength-to-weight ratio. The distinct morphology of 1D metallic nanowires, which includes predominance of low-energy crystal facets, long segments of smooth crystal planes and low defect density, makes them ideal candidates for potential applications. Those application include nanoreinforcement of composite materials [2,6–9], nanoelectrochemical systems [10–12], wearable nanoelectronics with distinctive sensitivity to force and electricity [13,14], nanopipette probes [15,16], and transparent flexible thin films [17–20]. The ability of 1D metallic nanowires to channel the flow of electrons or ions in one specific direction is a useful property for building blocks in nanotechnology. The behaviour of surface atoms depends on their coordination number. Those properties result in surface stresses, which vary depending on the type of nanowires and their crystal structure, and lead to high yield strength and mechanical modulus.

This review focuses on the synthesis methods and mechanical characterisation techniques of gold (Au), silver (Ag) and copper (Cu) nanowires. These Group 10 transition metals are sometimes called coinage metals. In Section 2, we review and analyse several synthesis techniques of coinage metal nanowires, as well as the nucleation and growth of heterostructures. In Section 3, we describe mechanical characterisation techniques. In Section 4, we discuss modelling of several measured properties via atomistic simulations and continuum models, as well as size effects in nanostructures. The final section presents perspectives of future mechanical analysis techniques and applications of metallic nanowires.

The experimental investigations on Au, Ag and Cu nanowires are sparse despite numerous potential applications. The ability of these nanowires to be functionalised and integrated into flexible electronic devices depends on their surface atomic arrangement and crystallographic orientation. For instance, the disfigured orientation of Ag, Au and Cu nanowires is mainly due to the generation of dislocation slip planes caused by the strain under tensile load while the compression load causes only partial dislocation slip. Recent studies have demonstrated the interplay between atomic arrangement and the output behaviour of by these single-crystalline nanowires with face-centred cubic (*fcc*) structure. It follows the ‘smaller is stronger’ trend, that is, the reduced dimensional scale of the nanowire increases the yield strength [21–25]. Here we review most of the available synthesis methods, mechanical and modelling studies of metal nanowires, and nanowire properties that arise from their intrinsic atomic arrangement.

Au nanowires have been extensively studied. They are widely used for improving thermal and current flows in various applications (nanoimprints of metal grids), for instance, in piezoelectric nanogenerators, roofing materials and brass furnishing. Implementation of Au nanowire as a junction in the interconnection of nanoscopic device components, which are subjected to the constant pushing or pulling forces, proved to increase the resistance of the system towards deformation while retaining the high strength of molecular device [29]. For example, a summary of experimental yield strength of high-purity Au nanostructures [30] is presented in Figure 1(b), where the stress measured for the yield strength is expressed as force per unit cross-sectional area. On this basis, it is crucial to understand how the early formation stage of coinage metal nanowires can improve their strength without sacrificing the ductility.

Ag, another precious metal, is a primary candidate for nanoelectronic devices owing to its low electrical resistivity. The use of Ag nanowires enables realisation of soft device systems, which can bear mechanical loads even during their fabrication and operation at room temperature as shown in Figure 1(c) [31]. The excellent mechanical properties of Ag nanowires make them a leading nanowire candidate to substitute the commercially available indium tin oxide (ITO) in a variety of loading-related optoelectronic and nanomechanical devices [32].

Cu has numerous applications such as storage media, catalysis, microwave absorption and clinical treatment, while being thousands of times more abundant compared to other coinage metals. Owing to their high stretchability that allows manipulation of reversible sliding characteristic, Cu nanowires are excellent materials for stretchable electronics [33]. Apart from that, when these potential features of the nanowires are structure-tailored to certain substrates using selective polymer molecules, the possibilities in developing better application of Cu nanowire-based stretchable electrodes become enormous. Further investigations on improving Cu nanowires have focused on improving their strength and ductility.

**Scheme 1.**
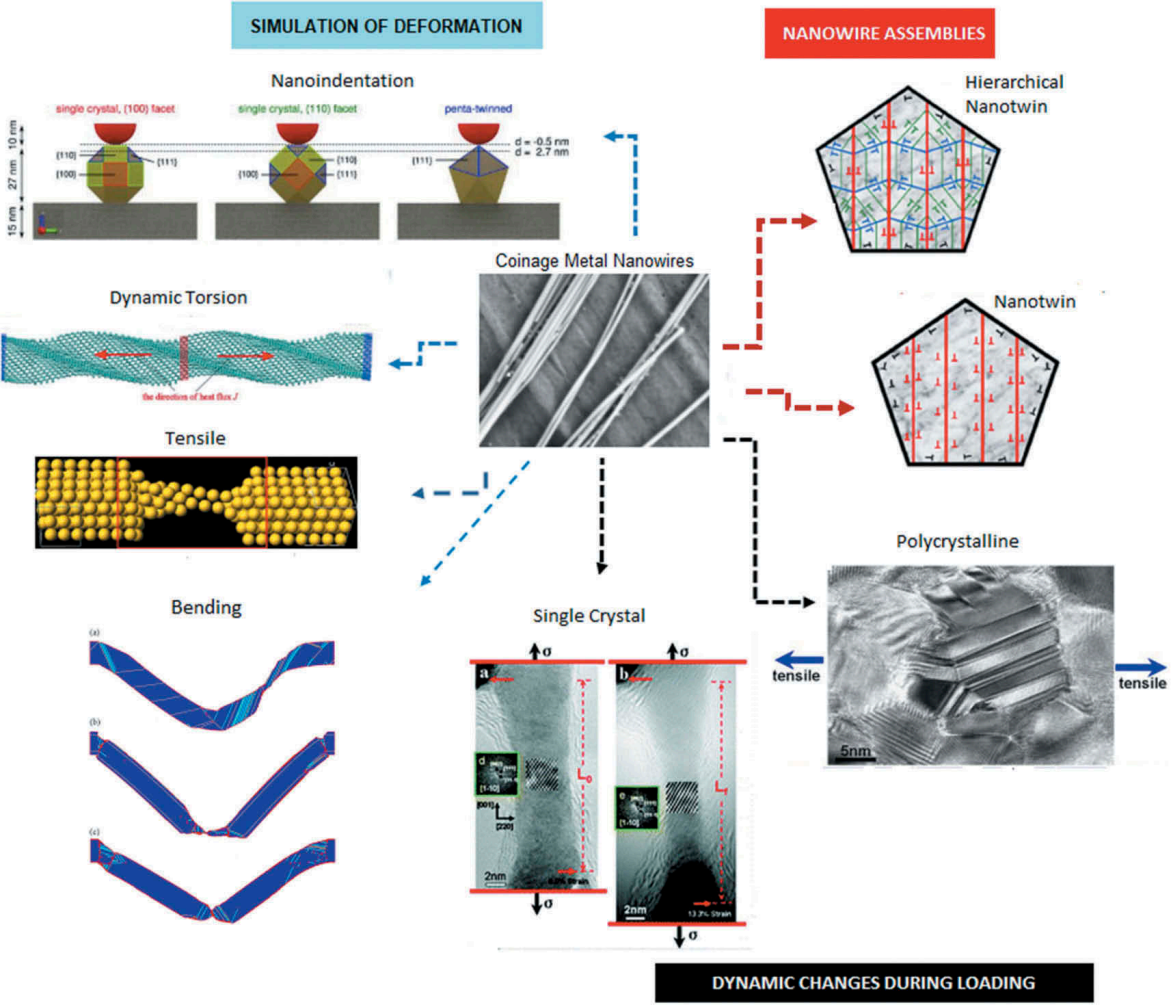
Synthesis, nanomechanical characterisation and simulation of coinage metal nanowires presented in this review (*reprinted with permissions from* [34] *licensed under a Creative Commons Attribution 4.0 International License, with permission from* [35] *licensed under a Creative Commons Attribution-Non Commercial-Share Alike 3.0 Unported License, with permission from* [36] *Copyright (2011) American Chemical Society, with permissions from* [37,38] *under IOP Publishing* [39,40], *are licensed under a Creative Commons Attribution 4.0 International License, reproduced from* [41] *with permission from The Royal Society of Chemistry*).

The described structures and their associated mechanical properties and modelling efforts evolved from advances in numerous fields that aim to link synthesis and physical properties with state-of-the-art performance in materials devices as summarised in Scheme 1.

## 2. Bottom-up approaches and solution-driven assemblies

Considerable efforts have been devoted to improve the quality of coinage metal nanowires, specifically at morphological control stage that stands as the premise for new nanotechnology building blocks [42]. Bottom-up synthesis technique which is the flip over of the top-down approach is preferable as it allows flexible alterations and promises a better opportunity to obtain unique properties of nanostructures. In the case of a bottom-up approach, the assembling of atoms and the clusters or the tiniest precursors of the metallic particles formed has a better chance to be controlled at a particular stage of synthesis until the required size of the nanostructures is accomplished [43,44]. Based on the established crystal growth structures, there are numerous bottom-up synthesis techniques used to produce 1D Au, Ag and Cu nanowires, the most widely explored schemes. Here, we limit the rest of this section to the description of a few strategies in exploiting the 1D coinage metal nanowires based on the solution-driven assemblies, namely

- Hydro/solvothermal synthesis and
- Template-assisted electrochemical deposition.

Apart from that, self-organisation seeds mechanism that involves the physical vapour deposition (PVD) starting from condense phase allows the growth of coinage metal nanowires of high purity with well separation of nanowires. Here, PVD on artificial cracks and directional solidification are presented.

The construction of hierarchical nanowires is a novel approach to improving the strength of coinage metals through the control of microstructure rather than modifying chemical composition. The synthesis methods should be able to control the initial and final size and morphology of wires and provide pure nanowires at high yield; they should be inexpensive and environmentally friendly.

### 2.1. Solution-driven assemblies

#### 2.1.1. Hydro/solvothermal synthesis

##### 2.1.1.1. Ag

**Figure 2.**
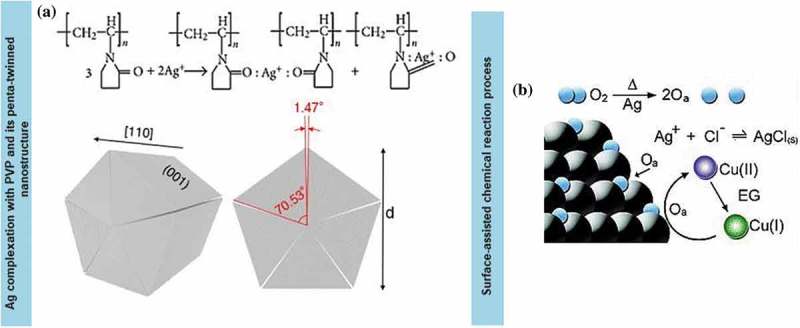
(a) Top: Schematic of Ag ions complexation with PVP chains. PVP owns polyvinyl skeleton structure with the strong pyrrolidone ring polar group. The polar groups (N-C = O) of the PVP chains interact with silver ions and form a coordinating complex. The illustration of 1D Ag-PVP coordination condition shows that high molecular weight (MW) PVP has relatively large silver ions coordinated along the long chain [72]. Bottom: Schematic of the formation of *fcc* single-crystalline subunits from fivefold-twinned nanowires [75]. (b) Schematic of the role of Cu salt in the surfactant-assisted soft chemistry process. The presence of molecular oxygen during the early stage of seeds formation allows absorbance and dissociation on the surface of the seeds [80]. (*Reprinted with permission from* [72] *under Creative Commons Attribution License* [75], *copyright (2013) American Chemical Society and reproduced from* [80] *with permission from The Royal Society Chemistry).*

Hydro-solvothermal synthesis for nanometre-sized metal particles in the liquid solution was initially introduced by Fievet et al. [45]. Reactions for the synthesis involved the dissolution of the ionic precursor, reduction in the solution, homogeneous nucleation and growth of the metallic structures within the solution. The famous method employed for Ag nanowire synthesis involved the modification surface of single crystal Ag nanoparticles using capping agent [46–49]. The method further became a fundamental reference for a reliable synthesis technique to optimise the Ag nanowires growth, which strongly favours nanowires with high aspect ratios. A high aspect ratio can be achieved by using simultaneous drop-wise injection and a mixture of a solvent, precursor and surfactant at a designated constant solution temperature. The most popular chemical reagent combination for Ag nanowires synthesis is the one pioneered by Lu et al. using ethylene glycol (EG) solvent, silver nitrate (AgNO_3_) precursor and polyvinylpyrrolidone (PVP) surfactant (Figure 2(a)) [50]. The surfactant not only hindered aggregation of nanowires, but also induced anisotropic growth of Ag nanowires. Various types of surfactants are also frequently used to assist and control the anisotropic growth of the atomic Ag nanoparticles to Ag nanowires that include hexadecyl-trimethylammonium bromide (CTAB) [51–53], sodium dodecyl sulfonate [54–56], vitamin B_2_ [57–59], polyvinyl acetate (PVA) [60,61], dextran [62] and polymethyl vinyl ether [63]. In most chemical approaches, the employed charged precursor contains ions of the desired metal [64].

Usually, both solvent and a reducing agent would act as reductant while the surfactant molecules would influence the interaction and reaction of the metallic element in the system [65–67]. For Ag nanowires, EG is usually used as a solvent in the solvo- or hydrothermal synthesis, as it enhances the reproducibility and scalability of the end product (Ag nanowires suspension) [68]. This occurs because heating generates much oxygen (O_2_) within the solution, which creates an autocatalytic effect owing to the high oxidation rate of EG to glycolic acid (GA, oxidation product) [69]. Then the newly formed Ag nanoparticles (the initial form of Ag nanowires) react with O_2_ at high temperature in the presence of an additional reducing agent (GA). GA is a strong reducing agent compared to other acetaldehydes. With continuous exposure to high-temperature surrounding and the aid from a surfactant, metals tend to crystallise into highly ordered symmetric face-centred cubic structures.

The strategies of hierarchy subnanometre granular structures and the refinement to nanograins can increase the strength of the coinage metal nanowires. The growth of Ag nanowires is derived from the hierarchical {100} planes [70,71] of multiply twinned particles (MTPs) from the self-seeding process, which yields non-uniform crystals. The MTPs with hierarchical nanotwinned structures can greatly alter the mechanical performance compared with that of the twinned bulk counterpart in other dimensionalities. For example, PVP molecules made the growth direction unified and produced a full coordination place around the Ag seeds. Besides, the long chains of PVP (MW ~ 800,000), added at a proper concentration, significantly improve the aspect ratio of Ag nanowires [72]. The higher the number of PVP carbonyl group within one PVP molecule, the more of coordinated Ag ions are attached, and the higher is the tendency for self-assembly into a 1D structure. Hence, a hierarchical assembly of relatively large Ag seeds is easily formed. Particularly, Ag nanowires imply that within the system, there coexist primary, secondary and tertiary twins and higher-order MTP structures in a single grain. Although the formation of nanoparticles is inevitable during the formation of nanowires, a selective scalable precipitation could solve these problems. The anisotropic growth assisted by growth agent through {100} facets results in their passivation while the bare facets of {111} are still active on the site. Specifically, the tendency of Ag^+^ to strongly interact with the lone pair electrons of an oxygen atom originating from polymer would also leads to a simultaneous reaction between the capping agent and AgNO_3_. Hence, the chemical potential of Ag^+^ would deteriorate during the nucleation process and thus increase the generation of Ag crystal seeds that form nanoparticles structures. Accordingly, the hierarchical MTPs formed, and the coordination, as well as the growth of 1D Ag nanowires with the aid of capping agent, generated the selective adsorption on the {100} lattice orientation facets. At the same time, the molar ratio between a repeating unit of polymer molecules in the presence of additional external Ag atoms increased the rate of formation of unconventional nanostructures via the spatial displacement [73,74]. Thus, the formation of unconventional nanostructures between nanowires with a high aspect ratio can retain their continuous network structures, which function as an electrical field path, while releasing the strain accumulated during stretching.

Such process usually generates coinage metal nanowires with fivefold penta-twinned structure, which is split by two {111} facets from a stacking fault arrangement. While the angle between the facets on the surface should be 72° (360°/5 facets), the measured value is 70.53°, which indicates the presence of lattice distortion of the atoms surrounding within the space filling direction (Figure 2(a)) [75]. The total deficiency in each nanoparticle leads to the lattice distortion that forms the seamless nanowires. The angular mismatch between the five twinned crystallites created gapless rods, thereby controlling the growth rate of nanowires and shifting their orientation towards {110}.

Nonetheless, surfactant alone will not be able to promote the growth of the nanowires as it is also essential to lower the surface energy difference between {111} and {100} facets. In the absence of additional seeding agents, the growth of Ag nanowires could be halted. The seeding agent would leverage the size of the initial nucleus, and the final size of nanowires fully depends on the type of the seeding agents used. Basically, the Ag precursor is reduced to form the Ag seeds at low concentration [76–79]. Later, generated zero-valent Ag atoms are selectively deposited onto the seeds that results in 1D growth at a relatively higher concentration in the subsequent step. For instance, CuCl and CuCl_2_ are used as the ideal seed agents for the growth of Ag nanowires [80]. The oxidative etching effect has not occurred even at a high ionic seed concentration due to the dissolution of oxygen during the reduction process. The decrement in the amount of dissolved oxygen during reduction reaction slows down oxidative etching and retains the high concentration of ionic seeds in the system (Figure (2b)). Therefore, the formation of Ag nanowires could rapidly accelerate in the absence of oxygen as the free ionic seeds promote the formation and dissolution of the nanoparticles [80–82].

Ag nanowires can also be grown by microwave-solvothermal synthesis [83]. The selection of seeding agent is crucial to ensure the success of the method. Apart from the Cl^−^ ions, Na^+^ can be used in the microwave-solvothermal technique [83,84]. The smallest mean size of the Ag nanowires could go down to ~45 nm with the length of wires up to 12 µm. Apart from Na^+^, several metal chloride have shown almost the same performance such as FeCl_3_, CuCl_2_ and KCl [42]. Cl^−^ limits the reduction of Ag^+^ to Ag°, thus, allowing direct growth under microwave radiation in the absence of the additional growth of new nuclei. Fe^3+^ and Fe^2+^ were also reported to show good control of the oxidative etching of Ag seeds [85]. Fe^x+^ ions with a higher charge [86] react with the absorbed oxygen on the surface, inhibiting the oxidative etching of MTPs. The oxidative etching induced the formation of tiny Ag nuclei which then grow into nanowires through the arrangement of individual atoms [84].

A similar process is applied to Au and Cu nanowires. Although they share a similar process flow, the resulting aspect ratio and other properties are different, as they depend on chemical reactants, reaction parameters, surface chemical conditions and dislocation behaviour.

##### 2.1.1.2. Au

Similar to Ag nanowires, significant work has been carried out to improve the synthesis of Au nanowires in the liquid solution. The most frequently used seeding agents in the hydrothermal synthesis of Au nanowires are PVP [87,88] and CTAB surfactants [22,89–92].

Generally, the aggregation of low-dimensional nanoparticles takes place during the assembly process to form 1D nanowires. When using CTAB, the reaction mixture does not require a temperature treatment but needs an acidic-containing solvent to achieve tuneable length and diameter of nanowires in the oriented attachment process. Thus, Au nanowires with diameters between 15 and 70 nm and lengths up to 12 µm were produced by a three-step seeding process based on self-assembly [93]. In this method, the first step involves a rapid reduction of an Au precursor in the presence of strong reducing agent, producing Au seed particles with a size range of 3–4 nm. The second stage requires the cooperation of Au seeds in the growth of Au nuclei through a slow reduction of Au salts with the help of seeding agent and surfactant [94]. The role of seed agent is to decrease the state of Au ion to meta-stable Au(I) from the *in situ* autocatalytic reduction that induces the increase of seed particles formation. The solvent-driven crystallisation entropy and reconstruction process minimises high-surface-energy facets and interfaces between nanoparticles, leading to the coalescence of nanoparticles and formation of a large crystal. For example, by using CTAB, the twinned crystal rods of Au with {111} orientation acted as specific surface passivation agent adsorbs onto specific Au facets [94–96]. In the third stage, the seeds grow into a single crystal nanorod. Self-assembly of CTAB molecules promotes the formation of longer rods. Interestingly, the capability of CTAB to form the spheroidal micelles can be accelerated by the addition of salts or co-surfactants to change their micellar behaviour during the reaction by the transition from sphere to the rod- or worm-shaped micelles [97]. During the process, salts or co-surfactants induce electrostatic repulsion between the polar heads of the surfactant molecules according to the Hofmeister series order (i.e. NaBr, NaCl and NaNO_3_) [98]. The Hofmeister order indicates the relative salt capacity to precipitate molecules in solution. The dimension of the short nanowire (rod) is controlled by adding small amounts of Hofmeister salts to increase the seed-mediated synthesis of short Au nanowires [99]. For instance, the use of oxalic acid to form nanochain of the Au networks depends on the ratios of Au^3+^/CTAB with oxalic acid concentrations in which accelerate the reaction. The reaction indicated the growth mechanism that is susceptible to crystal modifications which dominated by epitaxial micellar adsorption and adatom reorganisation [100]. As long as the appropriate quantity of other type surfactant depending upon the level of reactivity is added into the reactant solution, a continuous short range of Au nanowires will be formed. Additionally, the reactant parameters such as temperature, time, the solvent used and another type of additive affect the crystal growth of Au nanowires.

Interestingly, the chemical reduction of p-nitroaniline to diaminophenylene with sodium borohydride in aqueous solution proceeds six times faster in presence of Au nanowires that are stabilised by urea than by EG [23]. The higher amount of diaminophenylene is produced, from the reduction of aniline group, the higher is the number of corners and edges of Au metals produced on the surface of Au nanowires in deep eutectic solvents. As proven, eutectic solvents accelerated the formation of Au nanowire networks. Clearly, there is limitless variation in the combination of chemical reagent and reaction conditions used in the synthesis. Although variations on the chemical reagent used will produce similar structural output but with different properties. The same principle with different chemical reagents takes place for the case of Cu nanowires as further discussed in the next sub-section.

##### 2.1.1.3. Cu

Through the assistance of modified solvothermal reaction, it is possible to tune the speed of nucleation and growth of 1D Cu nanowires in a liquid system. To produce Cu nanowires, the reactant solution should be heated up to 300 °C in the presence of seeding agent [101,102]. PVP is a common seeding and reducing agent that prevents nanowires from becoming thicker as they grow longer. It is used for the synthesis of both straight and coiled structures. PVP would have selective adherence towards various crystal orientation facets, which should favour anisotropic growth of Cu nanowires. The introduction of PVA in the reaction solution would enable the formation of single-turn nanorings or nanobelts of Cu. The synthesis involves the dissolution of Cu ions and PVP in liquid medium upon continuous stirring. The principal driving forces of this reaction are the oxidative power of Cu ions, the reducing power of the reducing agent, the solvent and efficient manipulation of the reaction conditions.

The solvothermal reaction induced the formation of sliding planes in single crystals of Cu with relative low stacking fault energy. Hence, the residual internal stresses of the prepared samples can affect their final morphologies, resulting in perfect circular shapes of Cu nanowires. A wide variety of chemical reactant species, including surfactants, polymers, biomolecules and anions, could be employed for controlling the morphology and size of Cu nanowires, via preferential adsorption of particular species on particular crystal surfaces [70,101,103,104].

The hydro/solvothermal synthesis involves a direct chemical reaction of the precursors in solution. It produced metallic nanowires at high yield. The undesired by-products, such as nanoparticles or other nanostructures, are relatively minor and can be easily precipitated by centrifugation. Furthermore, this method does not require acidic or alkaline reagents for removing the synthesis template. Besides, the hydro/solvothermal synthesis is relatively simple and inexpensive, and its products depend solely on the types of the chosen chemical reagents. This approach is more attractive than the solid-state reaction (top-down method), as it guarantees mixing of the starting reagents at the atomic level, leading to a better control over the nanowire products.

#### 2.1.2. Template-assisted electrochemical deposition

##### 2.1.2.1. Ag

**Figure 3.**
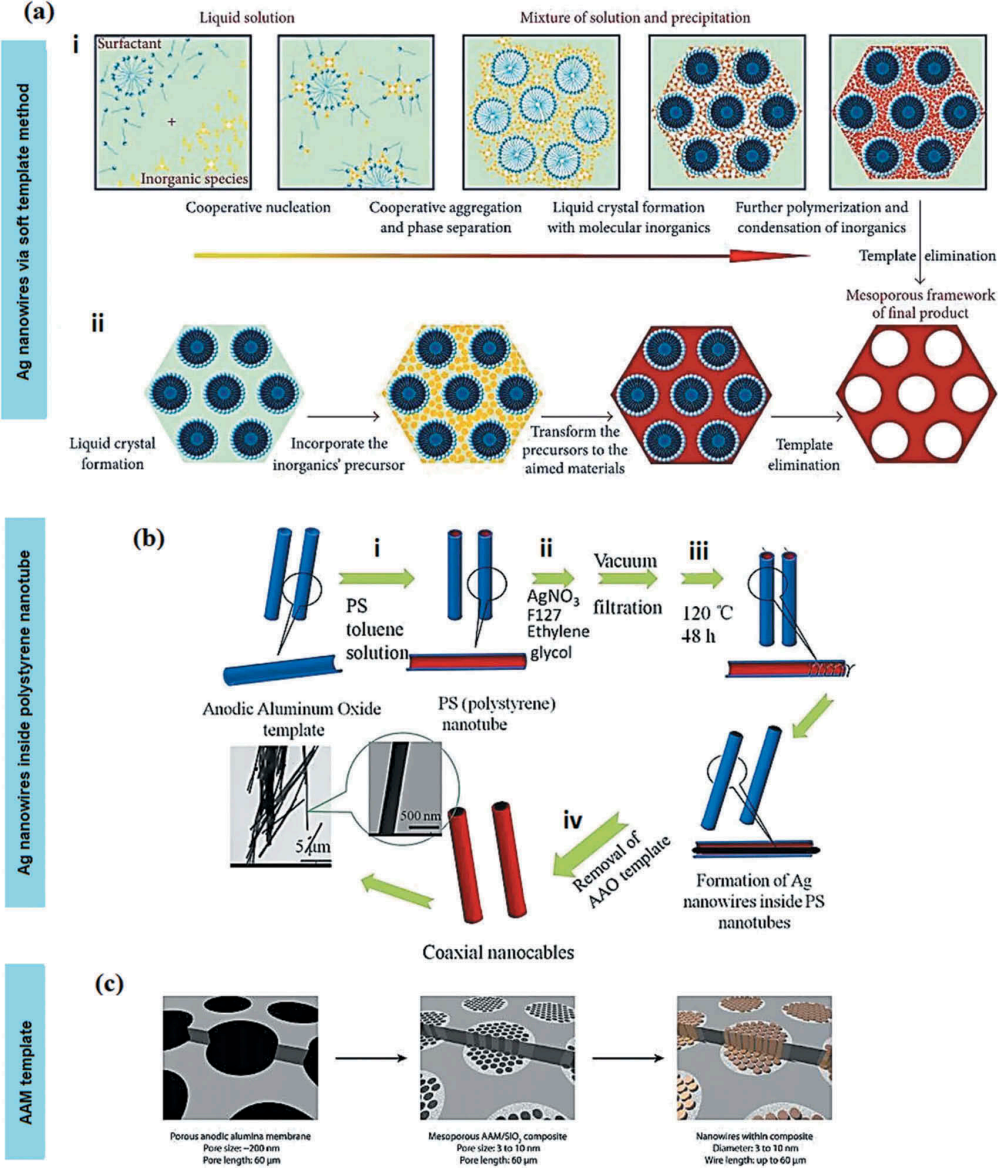
(a) Schematic of two soft-template methods of growing Ag nanowires. These two conventional procedures consist of polymerisation-condensation of inorganic (mark with (i)) and inorganic precursor incorporation that form the organic-inorganic phase as a product (mark with (ii)) [107]. (b) Schematic illustration of Ag nanowires inside polystyrene nanotube as a soft template and anodic alumina membrane as a solid template [113]. (c) The schematic illustration of the concept of nanowires synthesis within a composite. Keilbach et al. [108] chose the AAM pores as a template for the columnar nanowires. The columnar will be then used as the mould for the electrodeposition process for nanowires. (*Reproduced with permission from* [107] *Copyright (2007) American Chemical Society, reproduced from* [113] *with permission from Nature Publishing Group, adapted with permission from* [108] *copyright (2010) American Chemical Society).*

The composition of immiscible segments formed by co-block polymers has been widely used as the most applicable soft template-mediated technique for the synthesis of Ag nanowires. The initial structure (nanoparticles) formed from the surface-active agents and porous template materials aligned as the structural directing growth [105,106]. The method alters the morphology of the nanowires produced by mainly controlling the crystal nucleation and growth routes at each stage of template-directed growth reaction. The soft template usually formed from a surfactant or polymer and biopolymer. The formation path can be divided into three main phases as shown in Figure 3(a):

- Phase 1: Template preparation,
- Phase 2: Template reaction through the incorporation of synthetic approach including hydrothermal, precipitation and sol-gel reactions to produce the nanowires, and
- Phase 3: Template removal.

The soft template possesses broad potential as they have excellent repeatability and simplicity [109–112]. The use of template-based salt mixtures such as tetra propylammonium chloride (TPA-C) and tetra propylammonium bromide (TPA-B) offered much more adjustment in fabricating highly organised Ag nanowires. A liquid crystal of copolymer block is present before the addition of Ag ion. The copolymer with hydrophilic or hydrophobic aggregates formed the initial sphere micelles followed by the cylindrical micelles in the solution system. When the adherence of copolymer is sufficient, a pentagonal hollow array of liquid crystal structure steadily built up. The Ag ions, which were dissolved and reduced inside the solution, will precipitate within the cavities around the micelle rods by attraction reaction between the hydrophilic surfaces and hydration repulsion of the micelles. The Ag ion will then be reduced and solidified to create the Ag walls. Random assembly of rod-like copolymer interacts with Ag atom to yield two or three monolayers of encapsulated Ag at the outside surface of the micelles. The nanowires spontaneously assemble themselves into a long-range ordered structure (pentagonal packing). Usually, the transfer process experiencing adhesion problem at the interface between the substrate and the Ag nanowire electrode, which needs accurate transfer conditions to remain the quality and quantity of Ag electrodes in the medium. The difficulty of the transfer process includes the constraints in retaining the selection of substrate shape and large-area fabrication. If the polymer core layer remains after the process, it will significantly reduce the conductivity of the whole system; further downsides emerge from the use of a metal etchant and the waste of materials.

A new type of 1D Ag nanostructures, that is Ag nanocables (with larger aspect ratios and smaller line widths) has been synthesised using a combination of hard and soft templates growth methods (Figure 3(b)) [113]. The hybrid nanocable structure of Ag- polymer involve a complex process with hybrid organic reagents, where the system requires more than one different blocks or topologies [114]. The anodic membrane (e.g. anodic alumina membrane (AAM)) is the type of hard template used in the process to assist the uniform and ordered pores structure. For example, Ag nanocables wrapped in nanosheath polystyrene involves the channelling of metal solution into the void channels was through the self-assembly of Pluronic F127 and EG. F127 acted not only as the soft template but also as the guiding agent and reductant to the precursor used in the solution. The system is indeed highly conductive compared to other nanocables reported before. In the case of Ag nanotubes, the first hollow nanotube was created through the assistance of fatty acid (e.g. octanoic acid) [115].

Owing to the heterogeneity and its structural stability, electrodes made of Ag nanowires typically exhibit excellent electrochemical performance in a supercapacitor. For the electrochemical reaction, the improvement occurs at the interfaces between electrode and electrolyte. By reducing the path of the electron and improve the performance of active materials through the nanosized particles, improving the conductive network of the materials using carbonaceous matrix is improved, and the hollow or hierarchical structures could fit in high volume changes or the electrolyte which rapidly spread through into the inner of active materials [116].

Porous AAMs [71,117,118] and polycarbonate track-etched membranes [119,120] are frequently used as hard templates for the synthesis of Ag nanowires because their channel diameters and densities can be easily altered; they are also commercially available and easy to handle without breaking or tearing. The pore size in AAM template, such as Pluronic P123, is influenced by the types of electrolyte and the range of anodisation voltage during the reaction process (Figure 3(c)). The channel of the substrate is oriented perpendicular to the membrane surface to enable the build-up of Ag atomic layer which eventually induced the particles accumulation for the early formation of nanowires (nanorod). For porous membrane, chemical composition at the surface accelerates the diffusion. The AAM gives access to high aspect ratios of nanowires and permitted not only reproducible synthesis with precisely defined morphology but also with the precisely defined structure. The alteration of the synthesis condition such as the electrolyte salts increases the fraction of ordered pentagonal columnar over the pentagonal circular phase. In this case, the columnar elongates upon increasing of direct and under applied alternating current [121]. Further increase in the filling of these mesopores through the alteration of the electrodeposition parameter process (pulse or continuous deposition, thinning voltage and electrolyte concentration) led to excellent pore-filling uniformity with stable Ag nanowires inside the pore.

Current virtual electrode-solution interface process using classical double-layer theory permits digitised controlled electrodeposition system in a microfluidic device for the Ag nanowires synthesis [122,123]. The system employed a programmable light pattern to control the localised electric fields within the microfluid chip that promoted the local electrochemical deposition of free-standing nanowires. Specifically, the method involves a three-stage reaction:

- The reduction of Ag ion (Ag^+^) to elemental zero-valent Ag atom (Ag°),
- Comprehends the nucleation and growth of the reduced of Ag atom to form small seeds, cluster structures and,
- Delineate the particles growth stage, where the final Ag nanowires are formed.

The applied voltage and liquid conductivity control the final structural morphology of the as-produced thin double-layer film. The applied voltage typically set to 50 kHz, which is favourable for the formation of nanowires rather than compact electrodes of Ag.

The electrochemical redox reaction in the absence of dilute electrolyte using galvanic-cell supports is also possible for the formation of Ag nanowires. The galvanic support usually used to reduce the resistance of the solution. The Ag layer coated on the plate surface indicated the reaction took place on the galvanic support. As the concentration of the electrolyte reduced and getting low, the diffuse area of the double layer becomes significant with the layer formation thickness increase up to a micron depth. This induced the electro-migration of the ions towards the tip of the diffuse island layer in the direction of current flow. Therefore, nanowires develop around the sharp tip. As the electric field gradient increased, a massive accumulation of Ag ions deposition occurred near the tip than the ones close to the smooth or boundary area, which further contribute to the formation of nanowires. The larger the magnitude of the electric current field, the higher the density of incoming Ag ions within that region and subsequently, the tip of the layer becomes thicker. Lengthier Ag nanobelt could be grown in hard AAM nanochannel templates with dimensions depending on the AAM template [124,125]. Also, the biomass-derived monolithic activated carbon (MAC) template has been used for high yield and lengthier Ag nanowires through this method [126]. MAC is also known for its role as growth initiator surface, which commonly used for the formation of both nanowires and nanobelts within the system.

Furthermore, through this method, the formation of Ag nanowires could be accelerated through exposure of oxygen plasma under pulsed electrodeposition process [127]. The pulsed response helps in the fast formation of uniform and homogeneous pore filling within the template which assists by an adequate Ag ion concentration at the deposition interface.

Some of the significant factors influencing 1D Ag nanostructures are as follows:

- Ag ion species that form walls and their effectivity to promotes walls crystallisation,
- The concentration of surfactant (micelles, emulsion, liquid crystal and molecules) and molar ratio of surfactant to reaction precursor that includes their ionic intensity, pH and type of solvent used,
- environmental conditions (temperature, growth time, stabiliser), and
- controlling of product morphology (diameter, length and shape) [110,115,128,129].

##### 2.1.2.2. Au

The template growth involves the electrochemical redox deposition of Au nanorods inside a porous template, derived from a polymer thin film, through a track-etched template; it was developed by Martin [130–132]. Some of the well-known organic molecules or polymers used as a soft template for Au nanowires synthesis include 1-octadecene (ODE), insulin powder, PVP and octadecylamine (ODA) as listed in Table 1. Through the alteration of the aspect ratio of the initial nanorods, the length of nanowires can be changed as well [133]. For instance, Au nanowires can be synthesised using a lipid nanotube template with streptavidin that serves as a stabiliser. In this way, the organic template can be eliminated and particles can be linked using oxygen plasma [134]. The process involves the deposition of Au nanosized electrodes on the polymeric membrane via electrochemical plating reaction.

**Table 1.** Summary of template-assisted syntheses of Ag, Au and Cu nanowires (NWs).

Metal	Template	NW Diameter (nm)	NW Length (nm)	Ref.
Ag	PVP	80–130	>10,000	[157–163]
	AAM with gold nucleation layer	76	183	[127]
	Nanofibrillated cellulose	20	12,000	[164]
	Deoxyribose nucleic acid			
	AAM with PVP	45	200	[165]
	AAM with porous silica	30–40	200	[113]
Au	Oleylamine (ODE) and octadecylamine (ODA)	1.6	4,000	[157]
	Triton X-100 and Triton X-114	< 10	10,000	[166]
	Lipid nanotube 1,2-dioleoyl-sn-glycero-3-phosphoethanolamine (DOPE)	20	1,000	[134]
	Hydrogel of wrinkled polydimethylsiloxane (PDMS)	200–300	1,000	[167]
	Collagen (L-aspartate and L-lysine) Tobacco mosaic virus	30–70 2–30	500 300–1200	[168,169]
Cu	Track-etched polycarbonate membranes	200–400	10,000	[170,171]
	AAO membrane	15–200	5,000–25,000	[172–174]
	Copper salt in water oxidation catalyst	25–80	2,000–100,000	[175]
	Polyaniline nanotubes Pd-activated tobacco mosaic virus	250 24–46	7,000 >300	[176–180]

Apart from lipid nanotube template, porous silica template is of interest in the formation process of Au nanowire via electrochemical redox method [135–137]. This method was best understood for coinage metal nanowires, where the functionalised surface binds well to the depositing cation on the template pores through polymeric strands induced by aurophilic interaction [50]. Although the claims about the aurophilic interactions were more speculative, it is still a fascinating reported study in proving the formation of ultra-thin Au nanowires with high aspect ratios. Au nanowires with a high aspect ratio exhibit outstanding mechanical, optical, electrical and magnetic properties.

The high aspect ratio of Au nanowires formed by the electrodeposition template method (diameter less than 50 nm) resulted in a unique porous morphology owing to the assembly of small surface particles. A popular method to grow porous Au nanowires at high yield involves a filtration membrane where the residual Au ions deposited on Au nanostructures remain within the pores even after the rinsing stage [138]. Coarsening of the Au nanostructures results in the formation of solid Au nanorod segments upon further deposition of Au ions. The vertically aligned Au channel on the substrate cleaved the Au nanowires film and yielded individual Au nanowires that are independently interconnected with each other [139].

This approach involves a soft-enveloping function on the surface of the Au ion precursor immersed in mesoporous silica and a phase transfer method that takes place at the interface of the solid-liquid solution phase. The formation of Au nanostructures on silica composites with different morphologies and sizes prepared through different reaction time and temperature (i.e. goes up to 6 h reaction time). The Au precursor is initially distributed homogenously in the ordered mesoporous silica template. The formation of Au networks that develop from homogenously distributed Au ion precursors efficiently within a small space can be described by the classical LaMer curve [140]. The Au network formation comprises of three distinct stages:

- an induction stage,
- a nucleation stage, and
- a growth stage of Au nanowires.

In the induction stage, Au^x+^ ions are reduced to Au atoms. During this stage, Au nanoparticles are distributed over the entire silica template and separated from each other. Particularly, a liquid solvent which could not dissolve the Au ion precursor is employed as a block layer and is fully created to halt the Au ion species from moving towards the outer layer of a solid mesoporous channels template. Following the induction stage, Au atoms begin to form through a self- (or homogeneous) nucleation mechanism once the concentration of Au atoms reaches the maximum limit (supersaturation) point. On subsequent growth stage, a phase transfer mechanism takes place spontaneously across the interface of the liquid solution that typically contains the reducing agent ions and the liquid solvent which induce the reduction process at the interface between the Au electrodes and liquid solvent phases. The possibility for additional secondary nucleation to occur is none, and most of the reaction time is used only in the growth process.

It should be stressed that the mechanism of metal nanowire formation using a soft or hard template is complex and involves various chemical and physical routes. For example, if Au and Ni nanowire arrays are sequentially deposited using a nickel film on glass as a template, then the Ni array should be carefully removed after the growth [141]. Also, by increasing the applied voltage between the two electrodes, a gradual growth of the Au nanowire segment will occur owing to the increase in the current density. The wires had a length of tens of micrometres and tended to aggregate despite being horizontally aligned on a substrate [142]. There is a demand for a simple and inexpensive method of Au nanowire electrodeposition, which can be addressed by an electrochemical redox process through 3D cavities in a crosslinked gelatine layer that allows fabricating gelatine-templated Au nanostructures [143]. In this case, glucose additive is used in the synthesis solution to create the morphology of the Au nanostructures. Generally, soft- and hard-template methods have achieved good pore size and structural control. Nonetheless, research efforts in forming Au nanowires faced some limitations due to the difficulties to confine the soft nanowires structure within the template. Hence, some of the reported works are focused on Au-alloy nanowires since the dealloying finishing processes, which are believed could facilitate the formation of high-quality Au nanowires. These limitations are very critical for further development of these coinage metals since in most technological areas such as biomedicine and electronic devices, the requirement to have a well-defined morphology, composition and crystal structure of these metals to avoid averaging effects are crucial [144].

Relatively little work has been conducted using genetically engineered tobacco mosaic virus (TMV)-coated protein assemblies for direct and selective deposition of Au nanowires. The TMV template is known to create various assembly intermediates under a wide range of pH, including highly stable helices in acidic conditions [145]. The fabrication of Au nanowires involves the TMV-based platinum nanowires via electroless deposition of platinum precursors onto the outer area of the wild-type TMV disk. The tagged rods, which exclude from the recombination with wild-type protein, it then binds with Au nanorods, the initial structures after the nanoparticles formed and before it changed to nanowires.

##### 2.1.2.3. Cu

The morphology of nanostructure materials which are encapsulated by the closed shells of surfactant molecules varies according to the soft templates specification [146]. Anodic alumina oxide (AAO) membrane and polycarbonate membrane are frequently used as templates with polyelectrolyte coatings to grow and modify the surface charge of Cu nanowires. The AAO membrane has nanosized straight parallel pores, which have diameters of 300–400 nm, thickness of 50–60 μm and porosity of 0.25–0.50. The modification of surface charge in the membrane depends on the multiple layers of charged polyelectrolytes. The transient electrochemistry signals are quantified in Cu ion solutions of different salt concentrations, where Faradaic reactions dominantly authorised the Cu electrodeposition at the cathode and Cu dissolution at the anode. The most common approach in fabricating the cathodes is to deposit Cu or Au metals onto one side of the AAO membrane with the clamping approach. This procedure removes the initial distribution of the deposited metal that would influence the morphology of the electrodeposits [147]. In this case, polycarbonate membrane serves as a better template option compared to AAO owing to a simpler procedure of dissolving the template to obtain free-standing Cu nanowires. Additionally, polycarbonate membrane is more flexible regarding the template pore size selection, where the size can be made as small as 10 nm by controlling the etching conditions. Upon the deposition process, the Cu nanowires in polycarbonate can be directly integrated into the device fabrication depending on the properties obtained [148,149].

Besides the template method mentioned above, Cu nanowires can also be grown via electrochemical deposition in a scanning electrochemical microscope between metal ultramicroelectrode and oxide electrode [150–152]. Atomic contact was generated through the employment of another type of oxide electrode such as ITO encapsulated within highly ordered packed silica template with the vertical hexagonal alignment of small pore channels. The template which is also a promising candidate for hard-template casting [153,154] can be functionalised with organic groups [155]. The method generated encapsulated Cu nanowires with quantum conductance. Specifically, the Cu nanowires are fabricated through the electrochemically driven cooperative self-assembly of surfactant micelles and formation of silica by hydroxyl ions electrogeneration to catalyse the polycondensation of Cu ion precursor encapsulated around the amphiphilic molecules at the electrode or solution interface. A gap between two electrodes is connected through the electrochemical deposition of metal until they are linked and a nanowire is formed. The benefits of this approach are (1) the applied potential can be governed by means of a reference electrode in absence of mechanical deformation and (2) various arrangements and metals can be employed. Additionally, the electrodeposition tends to halt easily, producing atomic contact through the self-terminated process [150].

Basically, the Cu nanowires, similar to Au and Ag nanowires possesses polycrystalline microstructure in the initial growth level (<1 μm in length) and develops into highly dense coherent twin boundaries in the subsequent stage. They usually have a restricted capacity for lattice shift. As a grain boundary in a nanocrystalline Cu reaches a surface, it creates a groove. As a pair of coherent twin boundaries in a multiply nanotwinned Cu reaches a surface, they form a faceted zig-zag structure. The grooves and facets play different roles in chemical kinetic reactions. Typically, the chemical reactivity of Cu nanowires with pack coherent twin boundaries intercepting the free surface differs with the spacing of coherent twin boundaries. The smaller the coherent twin boundaries spacing, the better the properties. The Cu nanowires surface changes into a faceted structure of a very low atomic step density when intercepted by abundant CTBs. Understanding the nanoscale voiding kinetics becomes essential for controlling the void-free microstructure and the physical/chemical properties of nanomaterials.

A significant aspect of the template-assisted electrochemical deposition growth is that it can synthesise the nanostructures with an infinite variety of structures. The combination of versatility and different types of the soft template including organic molecules or polymers such as ODE, an insulin powder, PVP, ODA (Table 1), creates an infinite resource for 1D nanostructure development. The main result of this methodology was the new concept of a combine hard conductive core wrapped with the soft insulating shell.

The vast development in bioconjugation technologies permitted the use of ‘unfriendly’ biological molecules and this includes the famous rod-like plant TMV as an ideal soft biological templates for forming high aspect ratio Cu nanowires [156]. TMV ‘template’ is a useful biological template which is stable over a wide range of pH. TMV is a non-toxic plant virus structured as a hollow cylinder with a length of ca. 300 nm and the outer and inner diameters of 18 and 4 nm, respectively. TMV-templated Cu-nanorods were fabricated through a two-step electroless deposition process on immobilised wild-type TMV consisted of two stacked rings each of 17 subunits and helical rods. In the first stage, the surface of the virus template was activated by Pd nanoparticles on the surface. In the second stage, the Pd nanoparticles acted as an active site for selective catalytic reduction of Cu^2+^, leading to the growth of nanocrystal and the continuous formation of Cu coating on the template.

A method for the mass production of compound semiconductor nanowires that involves the direct reaction of component elements in a chemical vapour deposition (CVD) chamber is presented. This method results in nanowires, without the associated production of any other by-products such as nanoparticles or three-dimensional (3D) bulk crystals. Furthermore, no unreacted reactants remain mixed with the nanowire product in this method. This by-product-free nanowire production thus circumvents the need to tediously purify and collect nanowires from a mixture of products/reactants after their synthesis

Hence, it can be argued that through both the soft- and hard-template methods, the desired produced controlled density, spatial distribution, length, and orientation either horizontal or vertically aligned of 1D nanostructures through particular types of the substrate can be achieved to complement the constantly miniaturizing industries.

### 2.2. Physical vapour assemblies

#### 2.2.1. PVD on artificial cracks and directional solidification

##### 2.2.1.1. Ag

PVD allows to easily grow metallic nanowires of high purity. Note that despite their differences, both CVD and PVD allow to produce endotaxial structures, which indicate a fascinating event of growth. The endotaxy term refers to the growth formation of precipitate phases in a bulk matrix, with the formation of coherent interfaces surrounding the precipitate [181]. Conventionally, endotaxial nanostructures were synthesized using molecular beam epitaxy in ultra-high vacuum conditions of PVD approach [182]. The control on the position and morphology of Ag nanostructures is introduced in the form of Ag thin film cladded between the interfacial layer of GeO_x_ and SiO_x_ in the silicon substrate. Such control is difficult in the CVD process. The process involves the growth of symmetry-driven Ag nanostructures on a silicon substrate through annealing treatment (up to 800 °C) in air. The mechanism involved enhanced desorption of SiO_x_ and GeO_x_ layers at initial stages [183]. The Ag tends to diffuse into silicon through an interstitial-substitution mechanism during the annealing process [184]. The Ag nanostructures embedded coherently in the substrate and are quite stable system. Ag diffusion to reach the silicon surface has been enhanced due to desorption of SiO_x_ and GeO_x_. The process mainly involves low-temperature etching of intermediate oxide layer of the silicon/GeO_x_ or silicon/SiO_x_ substrate to help the formation of the endotaxial Ag nanostructures. In the initial cooling process, oxidation of the silicon substrate takes place which becomes a reason for increased thickness of the SiO_x_ layer, and later the condensation of GeO_x_ might occur at a lower temperature compared to SiO_x_. The morphological variation of Ag nanowires depends on the orientation of the substrate. The morphology of Ag nanowires is commensurate with the surface symmetry of the substrate either in twofold, threefold or fourfold symmetry for {100}, {110} and {111} orientations. The typical mean diameter and length of the Ag nanowires is in the range of 10–80 nm and 30–200 nm, respectively, with a wide distribution of sizes.

**Figure 4.**
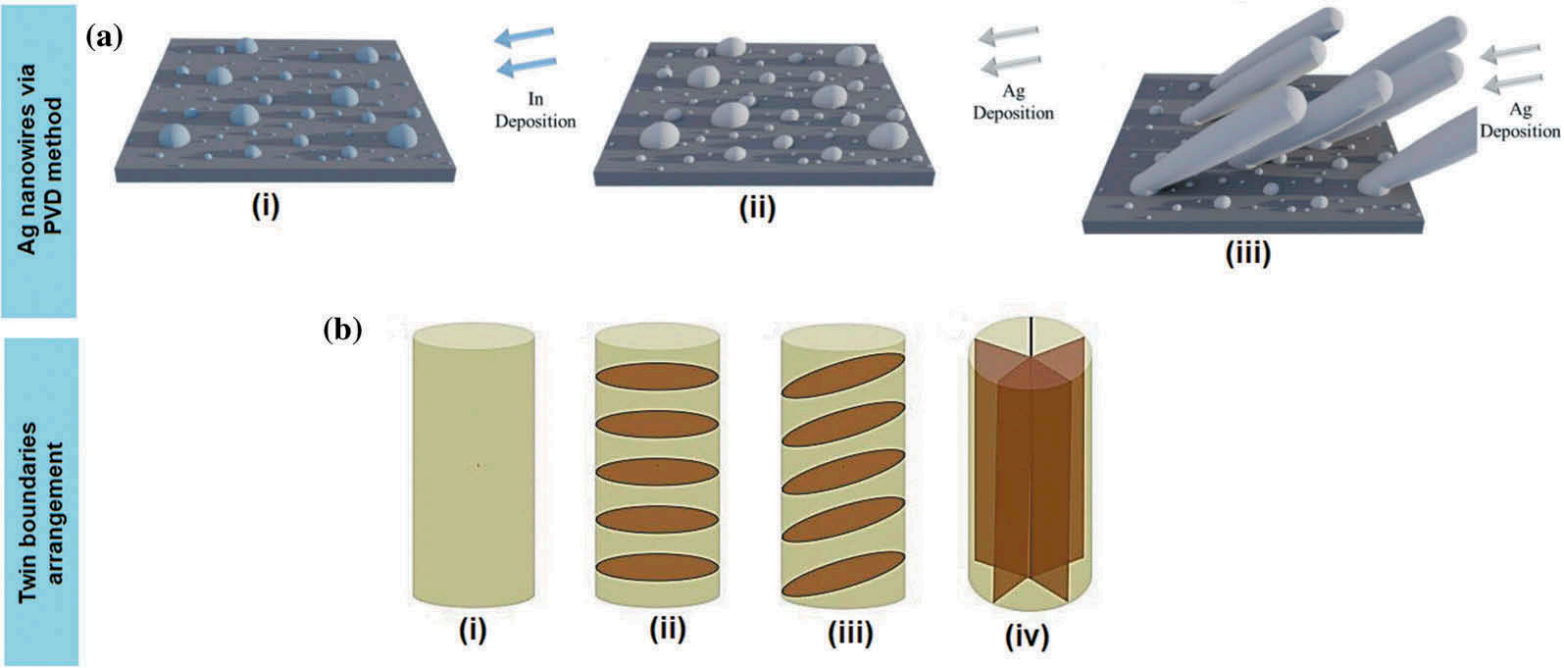
Schematic illustration from (a) on short Ag nanowires growth using PVD method deposited on the Si substrate [185] and (b) on the concept of the twin boundaries arrangement, coloured in brown with (i) single crystal nanowires without twin boundaries, (ii) horizontal, (iii) inclined and (iv) fivefold vertical twin boundaries [200] (*adapted with permission from* [185] *licensed under a Creative Commons Attribution 4.0 International License and* [200] *copyright (2015) American Chemical Society*).

Basically, the PVD deposition can involve no interruption of vacuum. Indium is used as a self-organised seed deposited onto a Si substrate and creates islands instead of a typical continuous film as a result of the non-wetting interaction (Figure 4(a)) [185]. Ag is then deposited and Ag atoms subsequently bind to the indium islands. This process is used for two reasons [186,187]. Firstly, the bonding of Ag atoms is stronger to indium than to the Si oxide layer. Secondly, more Ag atoms are deposited on the indium islands than on the Si substrate as a result of the geometrical shadowing due to the glancing angle deposition (GLAD) condition for short nanowires growth [188]. As a result, the vertical indium islands serve as seeds to differentiate the isolation of short Ag nanowires, and hence affect the mean diameter of short Ag nanowires. To separate the vertical indium components, it is important to deposit more indium atoms so only fewer indium elements remain in the vertical group. The isolation is a result of shadowing from adjacent seeds during the early deposition processes. The seeds that are fully shadowed receive very low flux and do not heighten. The mean diameters of the deposited indium islands are small enough for the further growth of nanowires.

A GLAD method is used with a combination of rapid switching deposition direction between crystal symmetry positions. The angle-switched GLAD can be practically expanded to form complex structures, such as 3D helical and segmented or core-shell metal heterostructures [189,190]. Such novel structures have significantly expanded the range of metal nanowires applications by exploiting various crystal orientations of nanowires.

PVD can yield coinage metal nanowires with tuneable length, hole dimensions and shapes; tailored wall composition; microstructure; porosity and structure. The nanowires are formed by tailoring the walls with controlled nanostructure with specific thickness (from a few nanometres up to several hundred). PVD offers the following features:

- Shell composition and microstructure: Although we focus on the growth of coinage metal nanowires (Ag, Au and Cu), the formation of other inorganic metal shell is also straightforward and effortless through vacuum deposition at low and mild temperatures.
- Substrate: The templates used for growing nanowires should be compatible with optical substrates, metal oxide thin films, silicon, metal nanostructures and flexible substrates such as polyethylene terephthalate.

##### 2.2.1.2. Au

Au nanowires can be grown within cracks of a thin film by PVD [191]. The cracks act as efficient templates because they are straight, tuneable down to nanometre size and can be easily aligned via controlled strain. The benefit of this method is that the Au nanowire forms concomitantly with macroscopic contacts [192]. The method involves mask deposition, crack generation, deposition of the nanowire material and lift-off of the mask. The Au nanowires are formed in cracks of the template thin film deposited on glass. The template is removed using an ultrasonic bath to obtain free-standing nanowires. This method has a limited freedom in the alignment of nanostructures. Also, only selected adsorbents and substrates that support self-organisation processes can be used, such as transition metal dichalcogenide crystals, Nafion, Teflon, amorphous fluoropolymers and polyamide films [193,194]. The artificial crack method requires a high degree of self-organisation approach that needs special materials in which the option for other materials with different functionality other than the self-organisation is restricted.

Directional solid-state decomposition in metal-Au eutectoid alloy system has been explored as a new method in synthesising ordered Au nanostructures [195]. The method employed phase separation through selective etching. The directional transformation of eutectoid alloys is a derivation of the eutectics directional solidification. In the formation of Au nanostructures based on the binary system, Au contributes as a minor phase (< 10%) in the eutectic-like reaction, resulting in a fibrous morphology. Interestingly, Fe-Au diagram is the only existing binary Au diagram that met the requirement in forming the Au nanostructures [196–198]. In the experiment, the bulk eutectoid alloy was cut into 1 × 1 × 0.1 cm pieces, which were dipped into the specific solution and left stirring for 24 h. The formation of reddish solution produced after the treatment was then characterised to obtain the total content of the Au and Fe elements. The produced structures in the form of fibril are not systematically distributed and are only partially well-ordered. The high portion of unorganised structures indicated that the steady state growth is not easily fulfilled. One main reason is related to the initial microstructure. The microstructure in the eutectoid phases is well known to be influenced by the condition of the initial structure and the level of homogeneity at the high-temperature phase. Hence, the solidification of that phase takes place. In this method, a range of Au nanostructures, including Au nanoparticles, short nanorods and nanofibres can be formed through this approach.

##### 2.2.1.3 Cu

PVD can yield high-quality single-crystalline Cu-based nanostructures with the thickness down to 1 nm, which include CuI nanosheets and nanowires. For example, CuI nanostructures with different crystal phases (*x* = α, β and γ) can be synthesised by PVD on a SiO_2_/Si substrate using *x*-CuI powder as the precursor and a noble gas (e.g. argon) as the carrier gas. The *x*-CuI nanostructures could growth on 2D transition metal dichalcogenides to form vertical heterostructures via a two-step PVD approach. The reaction starts with the growth of single-crystal metal dichalcogenides (e.g. WSe_2_ and WS_2_) on a silicon-type substrate using a PVD method. Later, the CuI nanostructures are grown on the as-grown 2D single-crystal metal dichalcogenides substrate. The *x*-CuI nanowires nucleate and grow on the top surface of single-crystal metal dichalcogenides, creating the vertical *x*-CuI/metal dichalcogenides. Briefly, for the synthesis procedure, a certain amount of CuI powder loaded in a small ceramic boat was placed at the centre of a horizontal tube furnace. The silicon-type substrate was placed in another ceramic boat downstream at a distance range of 8–10 cm away from the centre of the quartz tube. Before the reaction growth, the tube was purged with the carrier gas flow for several minutes at room temperature to remove the existence of oxygen in the reactor. The growth was stopped by turning the furnace power off and let the temperature of the system cool down to room temperature.

### 2.3. Summary

Comparison of synthesis techniques indicates that both the solution-driven and physical assembly routes allow much flexibility in the growth of Ag, Au and Cu nanowires. The capability of surfactants, precursor, reducing agent and seeding agent in solution-driven assemblies to assist in the alteration of metal nanowires surface combined with selective growth towards specific crystal facets, give additional advantages especially in tuning the morphological control and other surface properties. Still, a primary drawback from this method is the delineated accessibility of the particle surface (necessary for specific applications such as biosensing). Although solution-controlled methods tend to produce some aggregated nanowires, the dispersibility and properties of the nanowires can be enhanced through a post-functionalisation procedure. In some cases, several types of surfactants suffice to yield a stable and monodispersed nanowire dispersions. The nanoporous template, either as a pure metal, single-phase or two-component metal alloy has become imperative for better economic and latest improvised nanosynthesis technologies. Both the soft and hard templates are relatively a simple synthetic technique to produce a high yield of metal coinage nanowires and provide a high level of control over the wire dimensions and the composition either as a porous or solid nanowire. However, the growth of thin nanowires (<50 nm), beyond the range of template pore size, is still limited. As for industrial application, it seems that solvo/hydrothermal synthesis offers a better option, as the method can neglect tedious post-processing treatment involves high-temperature process in eliminating the synthesis residue. The use of temperature or acidic solution that needed in post-processing treatment of template method is quite incompatible with plastic substrate especially in the fabrication industry of flexible electronic devices. Hence, solution-driven assembly-based synthesis may have advantages and limitations depending upon the type of applications.

Orientation-controlled growth through the artificial crack and directional solidification via PVD method work well not only for coinage metal nanowires, but can also be extended to any material that can be deposited as a biaxial film. So far, PVD has been employed only at laboratory scale, using vacuum evaporation as an adjustable tool to obtain the structures. Additionally, the eutectoid transformation mechanism is non-cooperative and influences the limited uniformity and regularity of the obtained nanostructures.

Overall, there is still a great potential for the improvement in coinage metal nanowire syntheses. Their pronounced effects for Ag, Au and Cu nanowires, specifically for several nanomechanical properties, are reviewed in the next section.

## 3. Probing the nanomechanical properties

Recent advancements in experimental tools have facilitated the probing of nanomechanical properties down to single atomic chains [199–201]. General methods for probing nanomechanical properties of small-diameter nanowires (diameters of few tens nanometres and lengths of few micrometres) have been successfully developed in the last several years through a variety of techniques such as nanoindentation [202,203], tensile [204–208] and bending tests [209,210]. These quantitative tests often bring valuable insight into the atomic structures and potential development of the tested structures.

### 3.1. Nanoindentation

Ag, Au and Cu nanowires exhibit ultra-high yield strength and low hardening level as well as low ductility (tensile strength to fracture) [211–215]. Inadequate hardening is preferable as it can significantly affect the mechanical coherency of the constituent nanostructures in nanomechanical integration devices and other technological applications. The lack of decent hardening caused the absence of intrinsic obstacles within the nanowires, which halt the delocalisation of the crystalline defects to induce the macroscopic hardening [214]. Single crystalline nanowires of *fcc* metals such as Ag, Au and Cu distort via dislocation-mediated plasticity or deformation twinning and lattice reorientation without displaying noticeable hardening as indicated in Figure 4(b)(i) [200]. The horizontal (Figure 4(b)(ii)) and inclined (Figure 4(b)(iii)) twin boundaries are prone to migrate, causing twin coarsening effect. The twin boundaries play a significant role in ductility properties where low strain hardening is fully controlled through dislocations motion of the twin boundary barriers (Figure 4(b)(iv)) [216].

**Figure 5.**
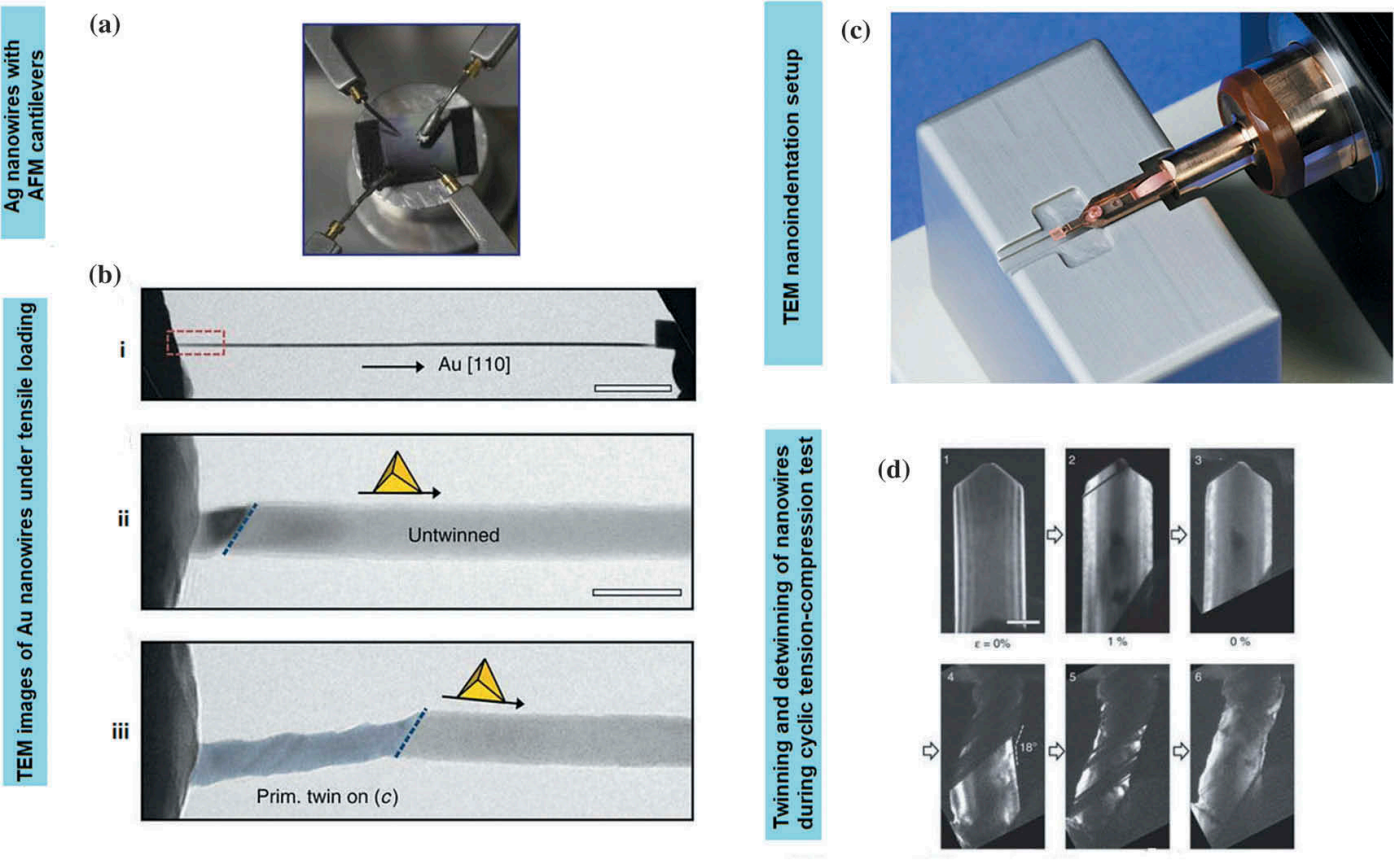
(a) Experimental setup of AFM cantilevers on the SEM stage [225]. The cantilever is mounted on a metal block of screw nut using a stable glue, providing excellent electrical contact with the close-up on the fracture area of the wire. (b) TEM images are used to monitor quantitative tensile tests before, right before and after breaking 20 nm diameter Au nanowires [4]. (c) TEM nanoindentation holder with the sample mounted on the metal rod. The image is originally courtesy of the Model 2030 Ultra-Narrow Gap Tomography Holder from Eden Instruments (http://www.eden-instruments.com). (d) Reversible compressive deformation through twinning and de-twinning transition during cyclic tension-compression. The first tensile loading near the tensile grip (image 1 to 2) and eliminated entirely by de-twinning during compression (images 2 and 3). The successive nucleation and extension of parallel nanotwins along the same slip area (images 4 and 5) with the accommodation of de-twinning during subsequent compression (image 6) [4]. (*Reproduced from* [225] *with permission from IOP Publishing and reproduced from* [4] *with permission from Nature Publishing Group).*

*In situ* indentation and retraction response are typically obtained from the scanning electron microscope (SEM) test [217,218]. The study on nanoindentation technique is mainly focused on ductility deformation process of nanostructures, particularly related to the determination of their hardening properties including the size-dependent properties of Young’s modulus [206,219]. Most of the studies evolved around in pertaining the plastic deformation of a single nanowire under axial loading for diameters less than 5 nm, that is, below the typical size of grown nanowires [220,221]. For instance, the smallest Ag nanowire diameter possesses higher strain hardening than the larger ones. It has been tested through nanoindentation with diameters ranging from 38 to 130 nm with the ultimate tensile strength of 4.8 GPa using a scanning electron microscope (SEM) at the strain rate ~10^−1^ s^−1^ [222]. The uniaxial tension of suspended nanowires using SEM was conducted using the actuator of nanomanipulator with the cantilever of the SEM as the load sensor inside the SEM. The ratio of force value over the corresponding instantaneous contact area yields the indentation stress. While the indentation strain is defined as the ratio between the immediate contact radius and tip radius. In this system, the nanowire is fixed to the tip of the nanomanipulator tungsten, and the process occurred with the aid of electron beam-induced deposition (EBID) of carbonaceous materials inside the SEM chamber [222–225] (Figure 5(a)). The nanowire is drawn away from the Si wafer, and brought close to the atomic force microscope (AFM) cantilever where it is then fixed on the side of the cantilever with the aid of EBID. Generally, the nanowires possess a linear elastic behaviour until a fracture occurred without considerable plasticity under repeated loading and unloading during tensile tests. Basically, Young’s modulus of most metal nanowires decreases with decreasing diameters where the softening trend is prominent in smaller diameter nanowire system [30,200,210,226,227]. The loading force-depth curve converted to the indentation stress-strain curve. Yu et al. [205] also presented a similar tensile testing technique that differs in the type of tips used. Tungsten tips show better functionality over other types in picking up metal nanowires during the process. Single-crystal and polycrystalline Cu nanowires with diameters of 500 nm show hardness values of 1.8 and 2.1 GPa, respectively [228], which are close to that of bulk nanocrystalline Cu (~2.2 GPa). Generally, the displacement during indentation is controlled by these two factors, namely, deformation in the sample and the bending of the nanowires caused by the high aspect ratio. Usually, a larger diameter tip (e.g. 500 nm to 5 µm) is used in order to correct the bending and thus produced only the bending displacement without indenting the sample.

*In situ* transmission electron microscopy (TEM) has been extensively used for characterising mechanical properties of nanostructures owing to its ability to directly visualise dislocations at the nanoscale. This method is particularly favoured in analysing individual nanowires with a small strain rate between 10^−3^ and to 10^−1^ s^−1^. Specifically, for fivefold vertical twin plane of nanowires, the obvious transition deformation occurs from slip dislocation to twinning under tension loading condition. While, the cyclic compressive-tensile deformation yields both compression and tensile deformations once the twinned nanowires were constantly twinned and de-twinned instead of being dislocated. For example, Au nanowires subjected to the tension force of nanosized punch showed the initial trailing of partial slip dislocations within the compressive strain areas [4]. In a compressive strain, the de-twinned deformation is primarily dominated in the structure (Figure 5(b)) [4]. The slip dislocation of the system is absent when the system is subjected to tension load. Instead, the twinned deformation of the structure took place when the nanowires are subsequently pulled into tension. For example, the twin boundaries of {110}Au nanowires annihilated the stacking fault on the twin boundary.

For nanoindentation experiments, a TEM holder (Figure 5(c)) is typically used in conjunction with a flat diamond punch for the compression test which facilitates the simultaneous acquisition of force-displacement curves which can be observed from the captured TEM images during deformation. The procedure of the process starts when the nominal flat diamond punch makes the contacts with the specimen that is normal angle to the imaging direction. Specifically, the nanoindenter is attached to a small transducer, which is coupled to a piezo tube. The hysteric behaviour produced from the piezo actuator is kept to the stationary condition during indentation process as to overcome the piezoelectric nonlinearities problems such as hysteresis and creep. The displacement signal output generated by the transducer directly measures the penetration depth of the nanoindenter, which can be programmed with a predefined displacement profile as a function of time to use the feedback system.

Micro-electromechanical systems (MEMS) are often placed inside SEM or TEM systems for *in situ* tensile testing of nanowires [229]. For example, incorporating a nanoindenter inside SEM [230,231] allowed precise control of load and displacement, yielding high-resolution force-displacement data from a silicon-based micro-mechanical device (MMD). Once the metal nanowires were released from the AAM, a micromanipulator inside the SEM was used to pick up a single nanowire to be placed between the sample stages. Later, the MMD was attached to an Au or tungsten rod with conductive glue to minimise charging effects. The setup was equipped with a nanoindenter, bearing a blunted diamond tip for manual indentation through coarse displacement of tens to hundreds microns. The nanoindenter was engaged under large elastic deformation, and continuously stretched the metal nanowire until its fracture (Figure 5(d)) [4].

**Figure 6.**
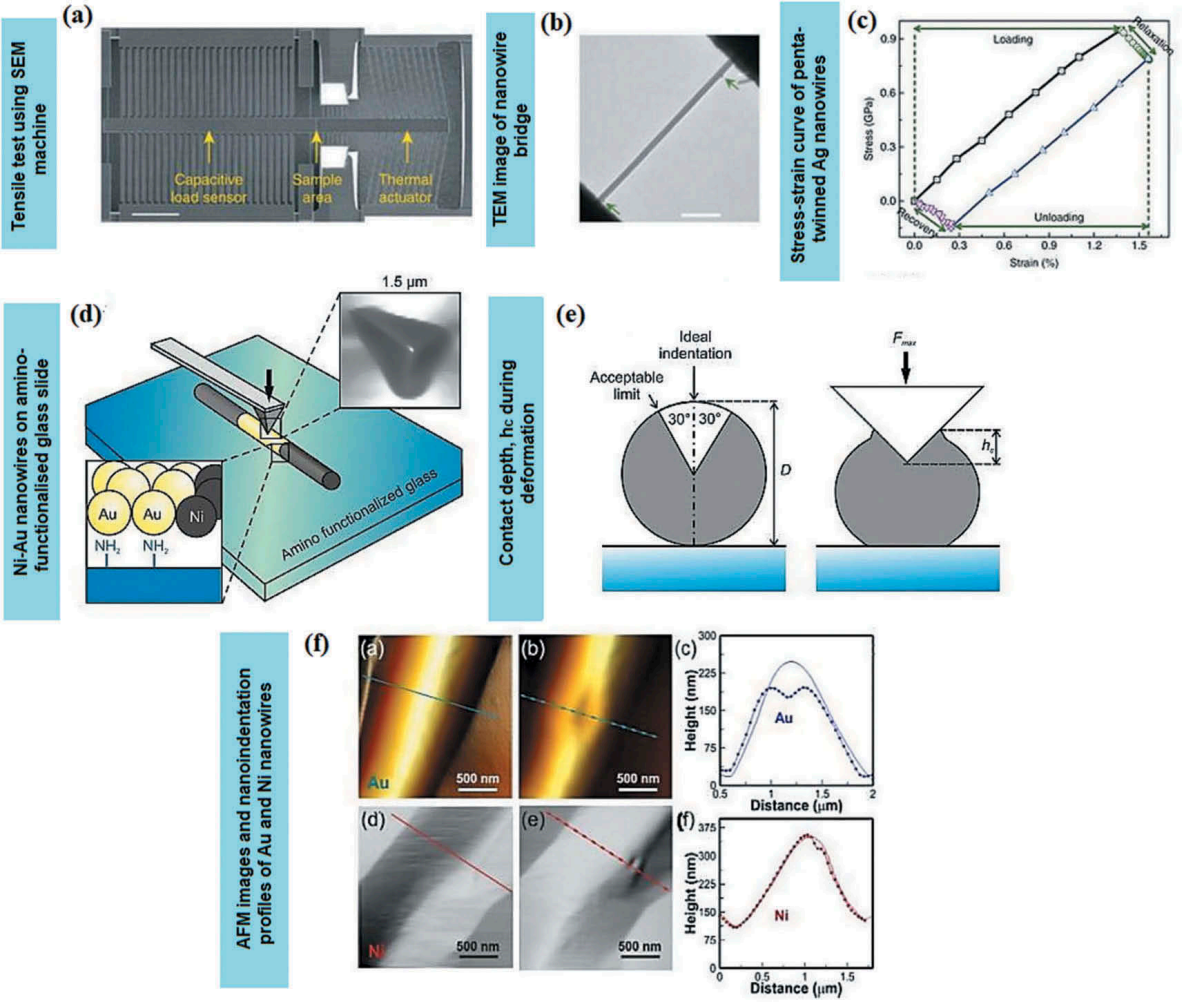
(a) The *in situ* tensile test of Ag nanowires using SEM with the scale bar of 200 nm [3]. (b) The low magnification of TEM image on the sample area of the nanowire bridge between the actuator and the load sensor with the scale bar of 500 nm [3]. (c) The stress-strain curves of penta-twinned Ag nanowires with a diameter of 120 nm. The relaxation and recovery states took 15 min to complete [3]. (d) Schematic of a bimetallic Ni-Au nanowire with the Au segment (yellow) attached to an amino-functionalised glass slide [236]. (e) Nanowire cross-sections indicate the acceptable indentation region (left) and the contact depth, *h_c_* during deformation (right) [236]. (f) AFM images and line profiles before and after the indentation test for both Au and Ni nanowires segments [236]. (*Adapted from* [3] *with permission from Nature Publishing Group and reproduced from* [236] *with permission from Elsevier*).

Nanoindentation is an efficient method to quantify mechanical properties of nanowires within the classical elasticity theory. The time-dependent deformation behaviour of penta-twinned Ag nanowires, which is related to stress relaxation for loading and complete strain recovery for unloading condition, has also been reported. The strain hardening test is performed using a MEMS-based testing system where the load is applied using a thermal actuator and is measured through the differential capacitive sensor on the other side. Carbon is used for displacement markers on the nanowires [3]. Both loads and displacement are measured simultaneously and accurately [232–234]. As the load on the nanowire specimen is decreased, the specimen elongation is increased. The temperature at the end of specimen is usually higher as compared to other areas as the stress is accumulated at the end of the specimen (Figure 6(a,b)) [204]. During the recovery process, the actuator is turned off, and the specimen is retracted to the initial position (Figure 6(c)). The stress relaxation typically emerged from the nucleation of initial partial dislocations created by vacancy diffusion. Meanwhile, the complete strain recovery occurred from the reverse movement of partial dislocations exerted by the repulsive force both from the twin boundaries and the intrinsic stress field of the fivefold twin.

Nanoindentation of crystalline metals exhibits a size effect that depends on the sample morphology [235]. This effect is extensively observed in AFM nanoindentation tests. In the experiment shown in Figure 6(d), a piezo-scanner indented both the bare substrate and the bimetallic nanowire until the desired positive force was attained. Each of the indentations performed should reach the maximum height of the nanowires. The offset opening is as far as 30° from the nanowire apex within the acceptable measuring contact depth, *h_c_* (Figure 6(e)). For instance, as the individual bimetallic Ni-Au nanowires are indented at each end and in the middle, the permanent deformation and pile-up surrounding the indented area occurred especially in Au segments as shown in Figure 6(f).

### 3.2. Nanobending

Crystallinity, defects and grain boundaries crucially affect properties of bulk metals. For instance, the strength of bulk metals can be increased via cold work hardening, precipitation hardening and grain-boundary hardening techniques which depend upon the restriction and hindering of dislocation movements [2,237]. However, the strengthening methods of nanoscale structures which rely on the similar concept of incorporation of impurities may be ineffective due to the facile surface segregation and expulsion. For quite a while, physicists and chemists believed in the concept of ‘the smaller is stronger’. That is, as the diameter of nanowires is reduced, and the yield strength or hardness increases accordingly. Thus, nanowires possess ultra-high strength compared to that of their macroscopic counterpart. As mentioned above, the high yield strength of nanowires stems from the presence of tiny particles that control dislocation activity. The microstructure control is one of the effective ways of controlling the mechanical properties of nanowire structures. Precise control of grain orientation and grain-boundary organisation within the penta-twinned nanowires account for their exceptional local strength and brittle failure. The principle underlying this theory is that the intersect grains within the twinning boundaries extend along the entire wire length to produce the homogeneous hardened structure. Free-standing coinage metal nanowires demonstrated grain-boundary hardening owing to the ability to fully control the grain orientation and boundaries within the structures. Therefore, observing and studying bending deformation or elastic resonance allow researchers to control quantum states in nanowires, which is useful for producing novel nanowire-based spintronic and photonic devices [238–240].

**Figure 7.**
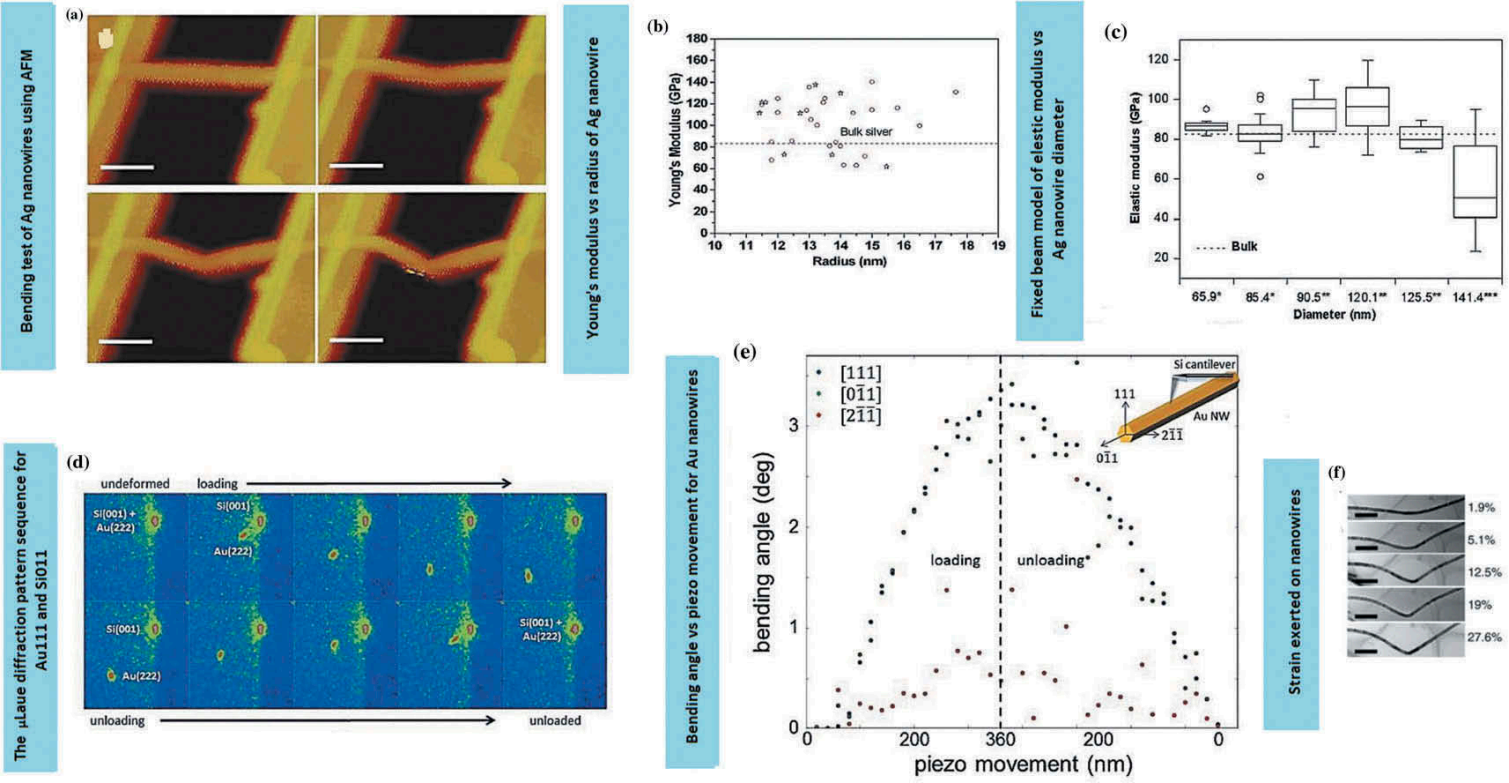
(a) The AFM images of a 23.6 nm Ag nanowire under the tapping mode test before and after the brittle failure occurred with the scale bar of 250 nm [241]. (b) The Young’s modulus plot as a function of Ag nanowire radius. The modulus remains the same before (circle) and after (star) the thermal annealing process [241]. (c) The plot of the elastic modulus vs diameter of the Ag nanowires with the nanowire diameter assigned from the fixed beam model (*), simple beam model (**) and simple fixed beam model (***) [242]. (d) *In situ* micro-diffraction pattern sequence for the Au 111 and Si 011 Laue spots evolution during the bending test [243] with (e) the bending angle plot as a function of the AFM cantilever movement [243]. (f) The snapshots of strain exerted on the nanowires [248]. (*Adapted with permission from* [241] *copyright (2006) American Chemical Society, reproduced from* [242] *with permission by AIP Publishing LLC, adapted with permission from* [243] *under the terms of the Creative Commons Attribution Licence and adapted from* [248] *with permission from Nature Publishing Group).*

The AFM lateral bending method permits measurements of various mechanical properties, ranging from elasticity to plasticity and failure [241]. The three-point AFM bending test involved the trench patterns of a substrate coated with 10–20 nm TiN film. Ag nanowires bridged the trenches through double-clamping at the trench edges using melted platinum. The phase transition from brittle to ductile occurred as a small displacement and elastic recovery took place. This was followed by the reduction in hardening as the grain boundaries slowly disappeared during the annealing which caused the plastic deformation. A dramatic increase in ductility was observed after 48 h annealing (Figure 7(a)) [241]. The Young’s modulus of the penta-twinned Ag nanowires with the diameter range of 22–35 nm is higher than that of bulk Ag (83 GPa, Figure 7(b)) [241]. The nanomechanical bending test has also been conducted using Nano R AFM equipped with a high-speed digital pulse frequency modulator (DPFM) electric control unit [242]. Typically, the results exhibit a deviation ranging from 0 to 6% (Figure 7(c)) [242]. Therefore, the values of elastic modulus are uncertain as they depend on the contributions of machine errors and possible defects present during the experiment. The shear defect along the localised stress contributed to the total nanowire deflection and plastic deformation behaviour.

Recently, the *in situ* three-point bending test is used to determine the elastic properties of nanowires carried out using µLaue diffraction [243]. The setup involves an AFM combined with a scanning force microscope for *in situ* nanofocused X-ray diffraction (SFINX). SFINX is compatible with various synchrotron end stations and can be combined with X-ray beams of sub-micrometre diameter. The setup allows both *in situ* imaging and *in situ* mechanical loading of individual nanowires to be carried out at the same time. The SFINX for nanomechanical testing was introduced by Ren et al. [244] and Cornelius et al. [245]. For example, the SFINX will bend the self-suspended Au nanowires where the deflection is measured based on the initial displacement of the detector point towards the Laue spots. The AFM tip was positioned above the centre of the suspended nanowire. The distance between the focused X-ray beams with respect to the nanowires is in the range of 16–18 µm. Allowing the bending and rotation of the nanowire enables the detection of crystal orientation of nanowires. The constant speed of the tip (5 nm s^−1^) pressing against the suspended nanowire deflected the structure. The µLaue diffraction recorded the piezo movement pattern, where the diffraction mapping sequence shows the reversible displacement of Au (111), and Si (001) Laue spots indicate a purely elastic deformation (Figure 7(d)) [243]. The overall output pattern of the piezo movement represents both the bending of the crystal of Au nanowire (inset) behaviour and the deflection of the AFM cantilever (Figure 7(e)) [243].

Since the 1920s, the limit of computed strain has predictably surpassed 30% of the upper limit of lattice deformation strain in metals [246,247]. The upper limit of lattice strain indicates the inelastic discrete dislocation activities, twinned deformation or stress-induced deformation phase transformation. Theory predicted that the continuous lattice shear strain could be as significant as 34.6% conserved within the confined crystalline lattice of metal nanowires [248]. The nanowires are bent or stretched by expansion or contraction of the continuous time Fourier series [249]. Deformation with continuous bending is observed via the snapshots of the time- and position-resolved atomic-scale imaging with the percentage of strain exerted on the nanowires usually in the range of 1.9% to approximately 27.6% (Figure 7(f)) [248].

Therefore, mechanical properties are crucial for ensuring normal operation and efficient reliability of coinage metal nanowires in flexible electronic devices. Table 2 presents a summary of quantitative *in situ* nanomechanical tests. Understanding the mechanical properties of these metal nanowires is essential for the successful development of such applications.

**Table 2.** Representative quantitative *in situ* nanomechanical tests for nanowires.

Nanomechanical test	Measured properties	Ref.
Magneto-actuation	Elasticity and ductility	[250]
Dual-beam focused ion beam and SEM/TEM using AFM cantilever	Tensile, resonance, bending, buckling, shear and compression	[199,251,252]
Thermal and piezoelectric	Stress and strain	[252,253]
Digital micromirror device in MEMS	Cyclic torsional and tensile fatigue	[254]
Peeling force spectroscopy in AFM	Adhesion	[255]
Nanoindentation in AFM	Hardness and elastic modulus	[206]
3D Bragg peaks using coherent X-rays	Evolution of strain (bending) and rotation for boundary conditions	[256,257]

**Table 3.** Calculated mechanical properties of Ag, Au, Cu and bi-metallic nanowires.

Metal	Loading	Twin orientation (°)	Grain size (nm)	Strain Rate (×10^8^ s^−1^)	Temperature (K)	Strength (GPa)	Ref.
Au	Tensile	-	2	4	-	~2.4	[276]
	Tensile	-	4.3	4	300	~0.6	[295]
Ag	Tensile	-	152	6	-	~1.15	[296–298]
	Tensile	-	14	6	500	~200	
	Compressive	-	3,000	^−^	298	0.056	
Cu	Tensile	90°	49.6	1	60	~181	[299–305]
	Torsion	120°	17.4	0.00009 rad.step^−1^	700	-	
	Tensile	30°	10.8	10	-	~5.1	
	Tensile	-	3.6	1	-	~4	
	Tensile	70.5°	40	2.9	300	~1.23	
	Tensile	30°	100	5	-	~3.8	
	Tensile	35.2°	25	1.2	-	~107	
Cu-Ag	Tensile	-	10	6	298	~969	[306]
Ag-Au	Tensile	-	10	1	600	~7.7	[307,308]
	Tensile	30°	4	2	800	~3.2	

### 3.3. Summary

The validity of the deformation mechanism of these coinage metal nanowires majorly depends on their crystal orientations. Such crystalline nanowires have impacts on nanomechanical properties owing to their ultrahigh flexibility and toughness. The reduction to the typical four-fold symmetry in coinage metal nanowire diameter prior to the fracture at large strain plasticity proved their high crystallinity. Generally, the high strain rates induced a continuous transformation of crystalline phase to an amorphous phase to give rise to a considerable change in a short-range atomic order and a disappearance of the shear elastic strain perpendicular to the tensile direction.

In this respect, a very close deformation progress can be observed directly and intuitively until the system reach their maximum conditions before a failure takes place. The trends and magnitude of the mechanical properties may give consistent values with the predicted ones. In this case, more descriptive method to measure the interactions between nanowires and the mechanism deformations beyond laboratories range becomes the subject matter. It should be noted that the extent and enlargement of the experimental zone beyond or below the maximum and minimum boundaries are of interest as the ability to accurately explain the dynamic response of the system is more desirable. Hence, more focus is necessary in the interpolation of refined physical continuum theories between the mechanical deformation and size effect. Several simulation approaches are discussed in the next section.

## 4. Modelling and simulation

Many of the complex geometrical conditions of nanostructures observed during growth are induced from the interplay of three fundamental processes: facets growth; droplet statics and the initiation of new facets. The incorporation of these process parameters into the numerical simulation model would be one of the efficient ways in obtaining insights that are not generated from the experimental methods. Given the additional inherent difficulties in producing a uniform aligned nanowires and geometrical growth of 1D nanostructures have not been fully realised, additional processing steps to achieve the desired nanowires assembly is necessary. Several simulation techniques can be employed for such studies. As the dimensions of the nanowire structures become comparable to the radiation wavelength *λ*, mechanical modelling and simulation methods can be performed rigorously using several available modelling and simulation analysis software depending on the desired outcome. The consideration of the laboratory data concerning the strength, strain hardening, fracture toughness, compression and strain rate sensitivity observations can be produced from mechanistic modelling. In this section, we reviewed several simulations and theoretical models in addressing the potential and reliability of exceptional mechanical properties of nanowires.

### 4.1. Atomistic and molecular dynamic simulations

Most theoretical studies on plastic deformation in single metallic nanowires subjected to axial load [221,258] are limited to nanowires with diameters below 5 nm. The small diameters of nanowires hinder direct correlation between theoretical and experimental results. The most frequent molecular simulation method that has been applied for nanostructures is molecular dynamic (MD) simulation [259,260]. MD simulation is mainly used as a method to measure the interaction between a set of atoms based on the integration of their finite motion equation. The integration involves Newton’s second law applied to each atom in the molecular structure based on the time discretisation. Most of the times, it is quite impossible to obtain an analytical solution that explicitly describes the atomic trajectories. Typically, the analysis involves several parameters that include the initial positions of atoms, which elucidated as the crystal lattice of the metal, while the initial velocities are granted according to the Boltzmann distribution at a particular simulation temperature. The MD studies identified two distinct mechanisms that mediate the structural response in noble metal nanowires: lattice orientation [261] and phase transformation [220]. These mechanisms are associated with reversibility and temperature dependence of structures as well as their pseudo-elastic behaviour. The MD simulation method becomes increasingly essential especially when it comes to the prediction of the embedded atom which could give a good approximation in the underlying interatomic potential of the nanostructures. The detailed understanding of energies and non-uniformities within the nanostructures is necessary. Thus, in turn, requires the models of the solid which should be both accurate and computationally simple. There are some common problems associated with the application of the simulations when the local environment of nanostructures is considerably different from the homogeneous bulk structure. The difficulties include surfaces, grain boundaries, internal voids and fracture characteristics.

**Figure 8.**
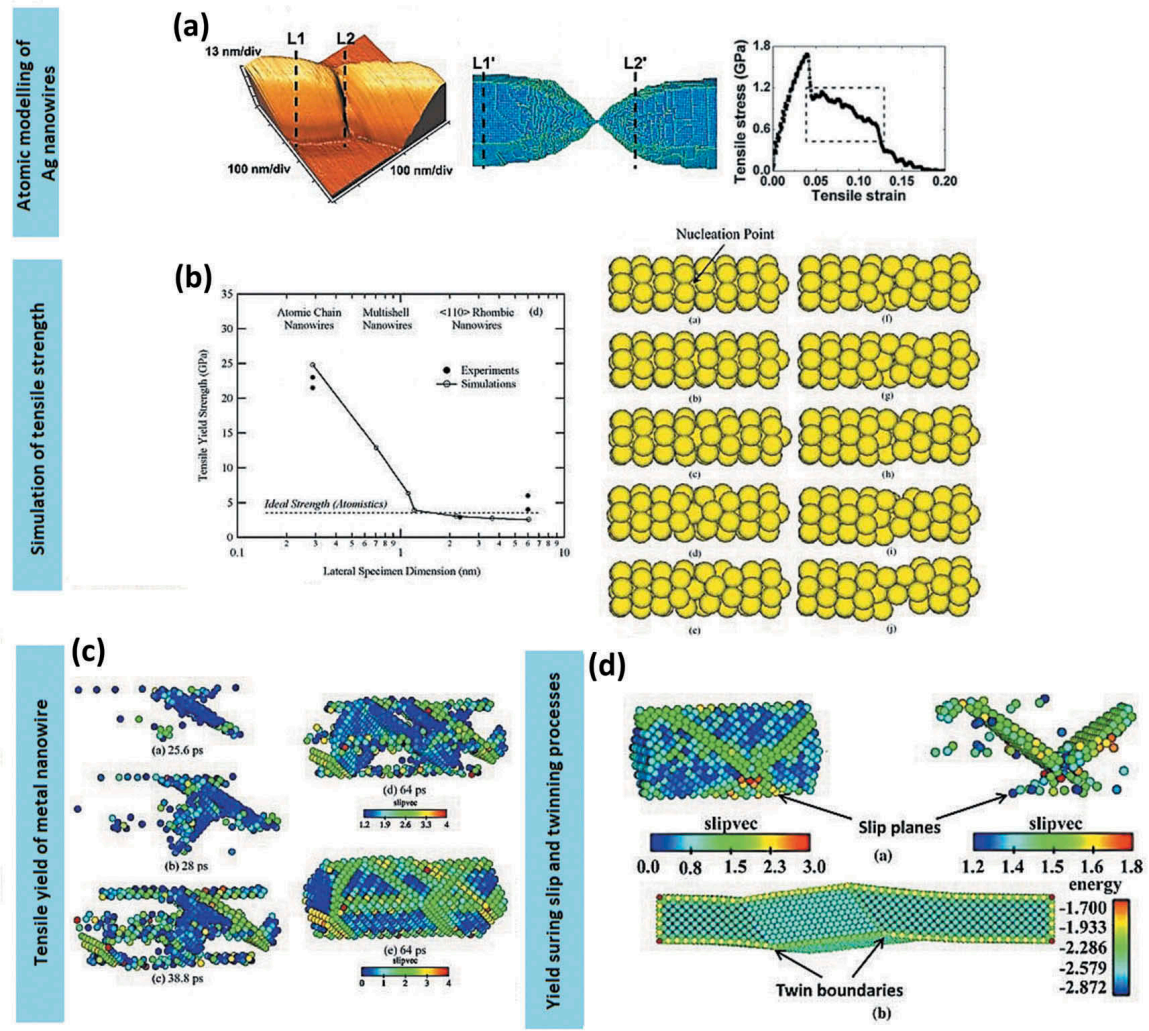
(a) The atomic modelling of Ag nanowire with a diameter of 15 nm under tensile stress before the deformation corresponds to the AFM image after the nanoindentation test. The tensile stress-strain plot shows prediction results from atomistic simulations [266]. (b) The multishell nanowire with the yield strength takes place at the local tensile fracture in the atomic junction [30]. (c) The tensile yield of the metal nanowires with the coloured atoms is defined according to the slip vector, and only the atoms that have slipped are shown in *a* to *d*, while all of the present atoms are shown in *e* [268]. (d) Slip during the yielding and twinning during deformation of the Au nanowire. The left snapshot shows all atoms, while the right snapshot presents only atoms on the slip planes. The atoms are coloured based on the magnitude of the potential energy [267]. (*Adapted from* [266] *with permission copyright (2008) by the American Physical Society, adapted with permission from* [30] *copyright (2004) American Chemical Society, reproduced from* [268] *with permission from Elsevier, reproduced from* [267] *with permission from Springer).*

The pair-potential approach based on density functional frames [259,262] also known as a semi-empirical embedded-atom method (EAM), under MD simulation methodology is commonly used for modelling of metallic systems. EAM has been used for more than two decades for nanoscale structures and has been validated to generate reliable results, as it offers a more realistic description of the metallic cohesion. EAM is based on the density functional theory and thus primarily aim in illustrating the strength and weakness of particular nanostructures [263,264]. The simulation starts with the initial velocities corresponding to the Boltzmann distribution at the simulation temperature. Later, the relaxation phase is carried out to permit the system to achieve an equilibrium state corresponding to the lowest energy state at that temperature. Finally, the tension or compression test can be exploited. The model for nanowires is typically extracted from bulk counterpart crystals according to the desired crystalline orientation that is similar to the method of top-down processing method. Through the pair-potential approach, specifically. However, the uncertainty of the volume dependence from the model is unavoidable. For an accurate approach, the results from first execution are usually precise. To achieve this, the use of a quantum chemical or density function method is important related to several parameters. The EAM parameters that usually requires for the simulation is based on the parameters of bulk structure (standard parameters for nanostructures measurement) that includes the equilibrium lattice constant, cohesive bulk energy, bulk modulus, vacancy formation energy, cubic elastic constants and diatomic molecule bond strength and bond length [265]. In general, the simulation for coinage metal nanowires start with the atoms placed in positions that mimic to the bulk face-centred cubic crystal lattice, with a pentagonal cross-section. The axial oriented nanowires along the {110} direction expand preferentially at the {100} lateral surfaces with the periodic boundary conditions were not imposed in any direction. The MD simulation then determines the deformation effect on nanowire size which merely focused on the surface condition during deformation. For instance, a 15 nm diameter of Ag nanowire under the tensile strain of 19.3% showed a final deformation stage before failure with the narrowest section comprising of 6–10 number of Ag atoms following the necking around the indented area of the original cross-section of the nanowire (Figure 8(a)) [266]. For nanowires of smaller diameter, MD exhibits similar atomic deformation process with the bulk ones but exerted onto a smaller size of neck limit. Based on the MD prediction, the 15 nm nanowire deforms elastically under the strain of about 4%, in which the tensile stress increases with strain up to the yield strength point. The simulation predicted serial occurrence of dislocations until failure (Figure 8(a)).

The application of EAM to a single atomic chain is controversial because it is not well suited to these extremely low-coordination systems. An argument is raised between the atomistic predictions of nanowire strength with the ideal strength calculated based on the bulk atomistic simulation. The quantitative result similarities are only achieved for diameter size range of 1–2 nm between the ideal strength of atomistic level and the predicted ones [30]. There is a counterbalance of strength enhancement from intrinsic surface stress restriction and strength reduction due to the availability of free surfaces and corners for defect nucleation, similar to the one observed in the bulk system. For example, the simulation yields local tensile bond fractured at the surface of multishell Au nanowire, followed by relative shear of surrounding atoms (Figure 8(b)). The sequence shows the contribution of atomic positions before nucleation on the wired peripheral near the centre, which then proceeds to the formation of a point-like defect on the wire surface and subsequently yield the adjacent shearing atoms of wire necking. The process indicates the differences between perfect atomic separation forced in individual atom chains with the dislocation plasticity in bulk *fcc* nanowires.

The two main key control factors in yielding the metal nanowires include the intrinsic stress induced by surface stress and different active slip systems for the propagation of dislocations. Typically, the magnitude of tensile yield stress is much higher than that of the compressive yield stress for small nanowires due to the effect of surface stress and different slip systems active in tensile and compressive yielding. For instance, the mechanical properties of Au nanowires strongly depend on the cross-sectional size [268]. The smaller the cross-sectional size, the stronger the nanowires as they could preserve their crystal structure during deformation. For instance, the yield strength of Ag nanowires of diameter from 6 to 1.4 nm increased from 4 to 4.4 GPa (Figure 8(c)). As the trailing partial dislocation nucleated in the middle of quad stacking fault at different strain rates, larger cross-sectional areas enhanced the chances of dislocation motion that lead to a decrease in both yield strain [221] and yield stress [30,210], with the stacking fault and surface energies enrolled in the fundamental deformation process. The orientations of partial and perfect slip dislocations governed the yielding of tensile property. The perfect slip formed by sequential partial slip occurs on the same plane. The trailing of dislocation in tensile caused the consistency in trailing dislocations under tension.

As for the *fcc*-type metal coinage nanowires [222,269], plastic deformation is usually found to initiated by the Shockley partial dislocation from the free surface condition. Slip and twinning can be understood by examining the influence of Schmid factors [270]. The lead trail of partial dislocations is nucleated from the surface or grain boundary [31], and thus, creates more partial dislocation on other slip planes, producing subsequent parallel stacking fault planes. The MD simulation using Large-scale Atomic/Molecular Massively Parallel Simulator (LAMMPS) code showed the occurrence of twinning that leads to partial dislocations nucleated on the plane right above the existing partial slip plane. For instance, tensile simulations were carried using LAMMPS MD at constant number of particles N, volume V and temperature T (NVT parameters) with the nanowire being elongated by 0.01% strain every 10 ps. The absence of a sharp decrease in stress reveals the presence of perfect nanowires. The difference between slip and twinning (Figure 8(d)) depends not only on the force type and heating level, but also on the geometry of the wires (different crystal orientations). The competition between partial dislocation slip and deformation is affected by material type [271]. For example, the axial orientation and side surfaces of Ni nanowires deform by twinning under tensile load (force parameter). Au nanowires are mainly deformed by twinning due to the heat at low simulation temperatures (< 5 K), and feature partial dislocation slip at high temperatures (> 200 K). The twinnability factors for Ni and Au are 0.878 and 0.828, respectively [271]. Different materials have dissimilar twinnability factors, according to theory of Tadmor and Bernstein [272]. The height of energy barriers, which are associated with deformation twinning and slip, is related to the twinnability factor.

**Figure 9.**
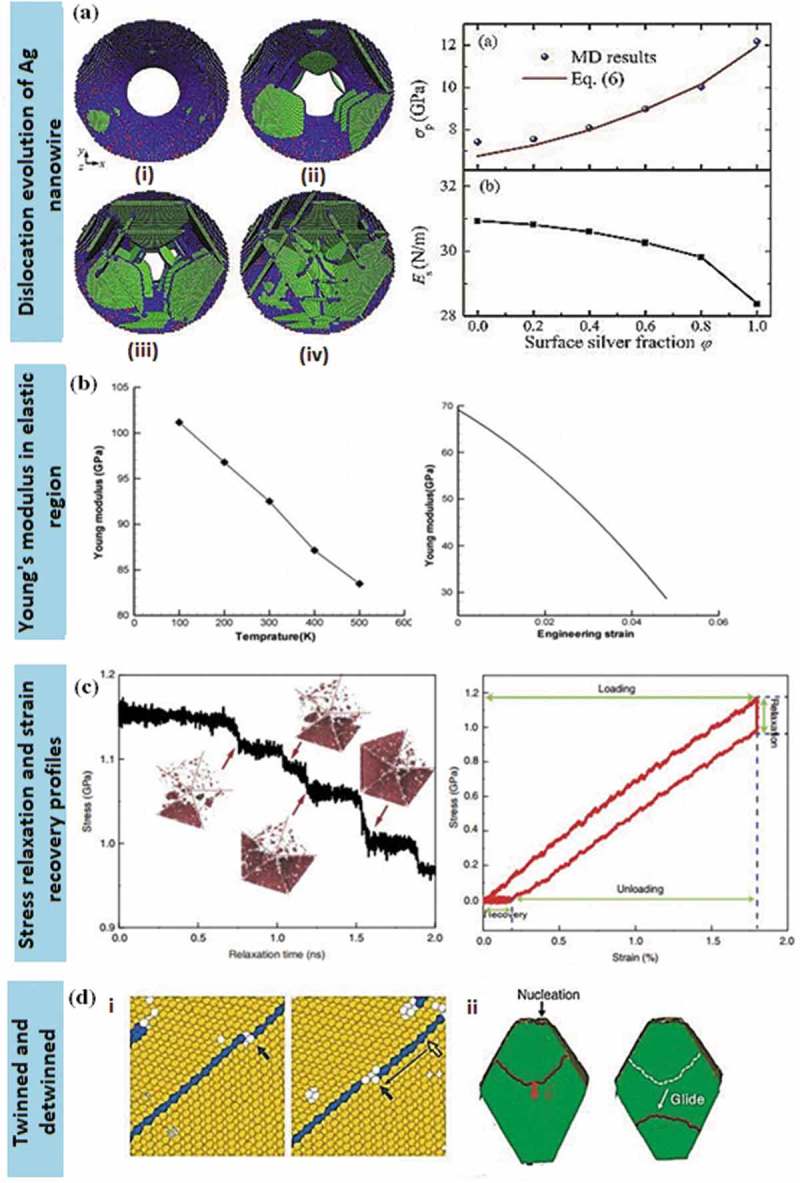
(a) The atomic model of Ag nanowires for the evolution of dislocation and their microstructure changed under different applied strain. The coloured atoms are specified according to the CAN method with the yield strength and their effective surface elastic modulus of Ag nanowires as a function of the surface fraction [279]. (b) The effect of increasing temperature and engineering strain on Young’s modulus inelastic region [280]. (c) The stress relaxation and strain recovery of the metallic nanowires [3]. (d) The twinning and de-twinning processes with the growth of a twin under tension and de-twinning by reverse glide dislocation with the same Burgers vector [4]. (*Reproduced from* [279,280] *with permissions from Elsevier, adapted from* [4] *with permission from Nature Publishing Group).*

For the structural re-orientation in nanowires with the diameters below 4 nm, large surface stress causes the phase transformation from *fcc* structure into a body-centred tetragonal (*bct*) structure [220,273,274]. The phase transformation is mainly driven by the surface stresses and allows the reduction in the surface energy of the wire. For wider wires (> 100 nm), a similar surface stress results in other structural transformation mechanisms that alter the crystal structure orientation. For instance, Gall et al. [30] proposed that *fcc* Au nanowires with the diameter less than 6 nm should exhibit a re-orientation from initial <100> direction with {100} side surfaces to <110> orientation with {111} side surface. A different scenario is observed for a bi-metallic system, in which the slip system glides along the planes and travels through the bi-metallic nanowire structure, leaving several stacking faults within the material (Figure 9(a)). In this case, the Au atoms show different atomic structures according to the atomistic defects in which green is used for hexagonal close-packed (*hcp*) atoms, blue is used for non-12-coordinated atoms and red corresponds to the Ag atoms using LAMMPS software. EAM potential is included where it used to describe the inter-atomic interactions. The Young’s modulus decreases as the ratio of surface Ag atoms increases. Bimetallic nanowires have a distinct surface modification potential, and thus can lower the residual surface stress and delay the formation of dislocations at the free surfaces. The Young’s modulus decreases linearly with increasing temperature. The mechanism involved relates to the minimum potential energy gained from the equilibrium distance between two adjacent particles. The balance of the repulsion and attraction forces between the atoms caused the exerted force to be zero. The equilibrium distance is an analogue to the elastic modulus. Increasing the temperature system will increase the distance of the particles at equilibrium. This causes the reduction in elastic modulus and results in a rapid progress of dislocation formation (Figure 9(b)) as well as quicker phase transformation towards plastic region. It shall be noted that Young’s modulus of the nanowires varying with the strain rate within the elastic region. The partial dislocation formation illustrated in Figure 9(c) within the twin boundaries of nanograins showed declined trend with respect to the discrete stress within a stress relaxation region. In this case, the atomic configuration of the simulated samples is 30 nm in diameter and 30 nm in length each comprises approximately 1.3 million atoms. Stress relaxation is a direct consequence of dislocation formation in the penta-twinned nanowires. The subsequent of unloaded forces yield a zero-stress state in which continuing relaxation produced a complete recovery of the residual strain. The deformation started by exerting the sample to the first stretch to a particular strain rate under the NVT ensemble, and later the relaxation process takes over under fixed strain. The MD simulation accelerates the thermal activation mechanism of vacancy diffusion. It is proved that as the nanowire diameter decreases, the relaxation time increases due to the size effects.

EAM simulations can predict the stacking fault energy and the surface energy through the implementation of Fire Dynamic Simulator (FDS) [275]. FDS optimised the entire structure where the lattice constant at designated temperature is homogeneously equilibrated using standard MD. The simulation setup comprises more than 4.4 million atoms for smaller diameter of thin wires, and with more than 175 million atoms for the larger diameter of the wire. Both the stacking fault energy and the surface energy caused the dislocation. The partial dislocation twinning glided downwards on the twin boundary (shown by white atoms). This resulted in layer-by-layer twin growth, producing the Burgers vectors of a/6{11$\overset{ˉ}{2}\}$ (Figure 9(d)) [4]. Meanwhile, de-twinning deformation glides through the opposite direction but within the layer-by-layer twin growth areas, eliminating one twin layer after the other via partial dislocation of the reverse glide. This posed a similar Burgers vector as the one produced from partial twinning dislocation.

The MD approach based on Sutton-Chen (SC) simulation is mainly focused on the size- and strain rate-dependent response, particularly for small-diameter nanowire systems (e.g. 2, 4 and 6 nm) [276]. The deformation characteristics observed include crystalline-ordered deformation, mixed-mode deformation and amorphous-ordered deformation. The utilisation of atomistic simulation to determine the strength level of nanowire geometry and the surface structure based on these characteristics proved the influence of the response outcomes [277]. For example, Au nanowires with a diameters smaller than 10 nm were successfully studied using the Nose-Hoover method [278]. The Nose-Hoover method is used to regulate the temperature to avoid any complex effects of the atomic thermal fluctuations in the NVT ensemble. The Au nanowire with a diameter of 3.85 nm intrinsically showed the re-adjustment of the atoms under the tension to minimise their kinetic energy. The length shrinks, the cross-sectional area increases upon relaxation, and the crystal structure is no longer *fcc*. The contradiction in the results typically related to the different stretching directions, and the dependency on the cross-sectional shape of the nanowire.

Modelling reveals important factors to be understood, which are mostly related to the optimisation of the intrinsic performance of nanowires. The optimisation was based on the re-construction of grain boundary, as due the particle migration mechanism is the primary focus for all the modelling studies described so far. The simulations particularly signify the hidden role of twin boundaries both during diffusion and gliding processes. The simulations also provide essential pieces of information regarding the phase transformation of desired physicochemical properties. However, the aforementioned theoretical discussions were limited to the fundamental properties of nanowires (e.g. grain boundary), while other complex properties are almost impossible to model analytically through the proposed simulations. Elucidation of the surface elastic effect, crack tip surrounding and other critical mechanical behaviours through continuum numerical methods has also garnered significant interest, and finite element modelling (FEM) is typically employed for that. FEM software has efficiently linked the fundamental conditions of bulk nanomechanical deformation to continuum failure scales. The importance and differences of the FEM simulation compared to the MD approach are discussed in the next sub-section.

### 4.2. FEM

Modelling of the size-dependent properties of nanowires is complicated because it requires high precision of parameters. The parameters should describe short and long nanowires, as well as various types of surface orientation and geometry. FEM can model 2D materials comprising multiple arbitrary finite planes of nanostructures with inhomogeneities and geometric nonlinearities of irregular surface structure. The Cartesian coordinates (*x,y,z*) are adopted in the simulation. The underlying aim of the method is to fuse the essential features of the individual single-scale simulations while edging the less desirable features. As mentioned in the previous sub-section, MD can increase the resolution of atomic-scale physics features from atomic to continuum systems. Computational efficiency where specific surface elements are taken into account as part of the measurement is attainable through FEM simulation [281].

**Figure 10.**
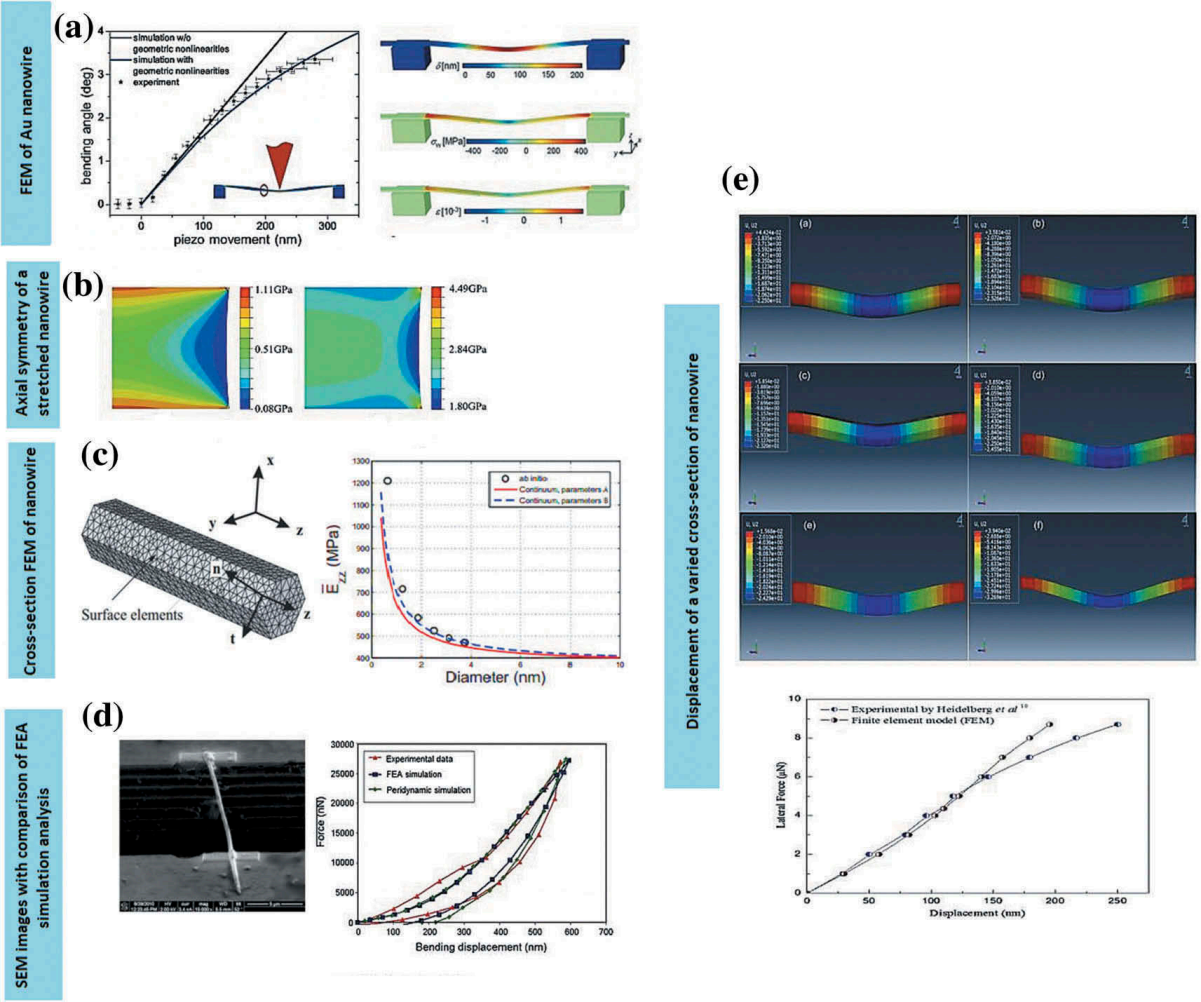
(a) FEM of bending angle with and without geometric nonlinearities of Au nanowires as a function of the piezo movement. Both the position and size of the AFM tip and X-ray beam during the test are illustrated. The illustrations of the total displacement, the stress *σ_yy_* along the wire and the volumetric strain for the gold based on the FEM simulation are presented accordingly [243]. (b) Radial stress shown in panel *a* and azimuthal stress in panel *b* demonstrate the axial symmetry of a stretched nanowire with a diameter and length of 6.66 nm [29]. (c) The meshing hexagonal cross-section FEM of nanowire specimen (left) and the diameter dependence of the effective Young’s modulus (right), which compares FEM and other models under prescribed stress [283]. (d) The SEM image of clamping nanowires with the comparison of finite element analysis (FEA) of nanowires against the experimental data and another simulation method [285]. (e) Displacement in various nanowire geometries (*a* – circular, *b* and *c* – hexagonal, *d –* pentagonal and *e* to *f* – rectangular) with the comparison of FEM results to the experimental data [287]. (*Reproduced from* [243] *with permission under the terms of the Creative Commons Attribution Licence, reproduced from* [29] *with permission under AIP Publishing LLC, reproduced from Ref* [283,285]. *with permission from Elsevier and adapted from* [287] *with permission under the terms of the Creative Commons Attribution License*).

Experimental data that can be analysed with classical beam theories using FEM are constrained to small wire deformations. Generally, the AFM bending test embedded with FEM simulator will have the maximum bending angle of 1° [282]. The displacement of the wire at the centre will be half of the wire thickness. Typically, simulation overestimates the experimental data due to the change of the contact between the AFM tip with the nanowire during the testing, and the deflection result of the lateral force applied. The minimum force to obtain the maximum bending angle depends on the total piezo movement. The calculated stress of the nanowires usually exceeds the bulk limit by a minimum of two orders of magnitude and that remains below the theoretical limit of ultra-high strength nanowires. For instance, the Au nanowire deflection is about 190 nm with the piezo movement of 280 nm along the stress *σ_yy_* axis and the volumetric strain field $\frac{\Delta V}{V}=\varepsilon_{xx}+\varepsilon_{yy}+\varepsilon_{zz}$ within the nanowire (Figure 10(a)). Therefore, the calculated stress along the nanowires exceeds the limit of bulk Au by a minimum of two orders of magnitude. It is still below the theoretical limit of ultra-high strength Au nanowires.

For a broad range of length distribution, the mapping of length-dependent deformation behaviour is useful for revealing the unique properties of the nanowires. The main characteristic, which is responsible for the high strength produced during deformation and is experimentally expressed by a virtual force (e.g. the image force), is the transition of constrained atoms to the formation of first partial dislocation. Typically, the FEM result will deviate significantly from the MD. The ‘two-phase’ FEM flow is applied where the matrix and the fixed atoms are treated as constituents that have different shear modulus. The screw dislocation in an infinitely long wire builds up from the fixed atoms; it is then evaluated regarding the distance between the fixed atom and the dislocation and the distance between the bottom atoms. In this case, the Burgers vector and shear moduli employed in the model explain the dislocation formation due to the shear stress neglecting the force. For example, FEM simulation of 3.53 nm Au nanowires proves the presence of multiaxial stress and large stress gradient with the maximum stress accumulated at the corner of the tip from the simulation results (Figure 10(b)) [29].

FEM is also used to solve the surface elasticity of the nanowires where the classical matrix and vector forms of the domain are discretised into volume elements [283]. As noted before, the surface free energy is larger in nanowires than in nanoparticles. The surface stress is important in theoretical modelling as the continuum theory of nanowires involves the second derivative of the effective energy derived from the strain gradient element [284]. For example, the FEM created a standard diameter and length of *L* = 4*d* for nanowires indicating different meshes with increasing element densities for the production of first relaxing radial strains (Figure 10(c)). The interpolation of field displacement is obtained from the FEM linear shape function based on continuous radial displacement and also the primary radius of the nanowire.

Further elastic-plastic bending behaviour of a finer mesh nanowire is possible to achieve since the FEM simulation permits better balance in terms of accuracy and computing time by using ANSYS system. The properties of tetrahedral nanowire such as plasticity, stress stiffening, large deflection and substantial strain capabilities are more accurate as compared to the experimental reading at some point. The nanowires are clamped at both ends to established zero-displacement boundary condition in all axes (*x, y* and *z*) [285]. The bending deformation obtained at specified nodal displacement at the midpoint of the nanowire and the displacement involved increased linearly through several time steps. The displacement applied at the midpoint node in every time step and the total cycle of step time during the loading is deduced according to the laboratory data which typically varies between 13 and 22 cycles for Ni nanowires. The reaction force calculated at the support points where the force applied is maximum at the midpoint based on the simulation. The loading and unloading curves for various combinations of elastic modulus and yield strength nanowires were defined clearly by ANSYS through the comparison of experimental and the best correlation fitting data. For instance, the 80 nm tetrahedral cylindrical Ni nanowire provides more than 30 segments along the nanowire cross-section. FEM of four independent bending experiments predicted the variation of elastic modulus to be between 190 and 255 GPa (Figure 10(d)). Meanwhile, the yield strength varies between 3.1 and 4.7 GPa. Clearly, the yield strength of nanowires is typically higher than that of bulk material due to a lower defect density [210,286].

According to the beam theory, the circular nanowires at the onset of the plastic deformation demonstrated the effects of flexural rigidity and deflections in the elastic region at the maximum displacement. In this case, FEM involves a two-step method to determine the stress-strain curve of the cross sections:

- Linear elastic, and
- Non-linear analyses based on ABAQUS modelling.

The first step known as linear behaviour stage involved the small force displacement that requires the ’OFF’ set to activate the non-linear geometry (NLGEOM) effect. During this stage, certain eigenvalues of Young’s modulus, Poisson’s ratio and density are used. Meanwhile, the second step needs the ‘ON’ set to obtain the non-linear behaviours. In a plastic region, the isotropic hardening with the eigenvalue of yield stress is used. Usually, FEM simulations of elastic deformation and the load-displacement curve agree well with the experiment. There are three main reasons behind the small differences between the results. First, swinging or slipping error may occur during the AFM testing. Second, the displacement influences the loading position at the mid-span of the nanowire that is beyond the SEM resolution. Third is the presence of pores and defects in the loaded samples. Consequently, circular Au nanowires showed the lowest deflection, followed by hexagonal, pentagonal and rectangular nanowires (Figure 10(e)) [287].

FEM provides an approximate solution to boundary value problem through the implementation of partial differential equations. In this case, COMSOL Multiphysics software is used to simulates the model, meshing and execute computations [288]. In the simulation, the whole constituent geometry is divided into small regions of a simple shape that have finite elements and interconnected points known as nodes. The various methods are then used to solve the problem based on the system approximation condition in the presence of local shape function for finite elements which subsequently could lower the associated error function. As previously mentioned, the physical properties of the bulk counterpart such as thickness, Young’s modulus, Poisson’s ratio, shear modulus and density are assigned to the elements. However, a simplification to a smaller dimension than 3D is not possible with this method due to the real-world boundary conditions of breaking symmetry. The homogenisation Monte Carlo (MC) method applied to the transport and reactions near the surface of nanowires allows to compute the charges of microscopic particles in the boundary layer. Nonetheless, the charge-transport problem still exists. In the following sub-section, MC simulation and other parallelisation techniques are described in detail.

### 4.3. MC simulation

The study on the dynamic elongation of nanowires is one of the critical parameters in building the sophisticated molecular nanoelectronic devices [289]. The understanding of electron transport through metal-molecule-metal junction configuration is critical in order to relate the bonding structure with the elongation behaviour of nanowires for a particular system. Few critical parameters such as the number of nanowires, the temperature of the system and the area of the substrate are included in the system. The constraints such as volume constant and number of atoms are automatically included in the periodic boundary condition simulation. A small random displacement of atom within the nanowires yields an intrinsic change in the potential energy function. If the resulting change in energy is positive under the influence of heat, the Boltzmann factor is considered to contribute to a random number for a new random configuration. Meanwhile, if the resulting change in energy is adverse, the displacement of the atom is considered as a new configuration.

**Figure 11.**
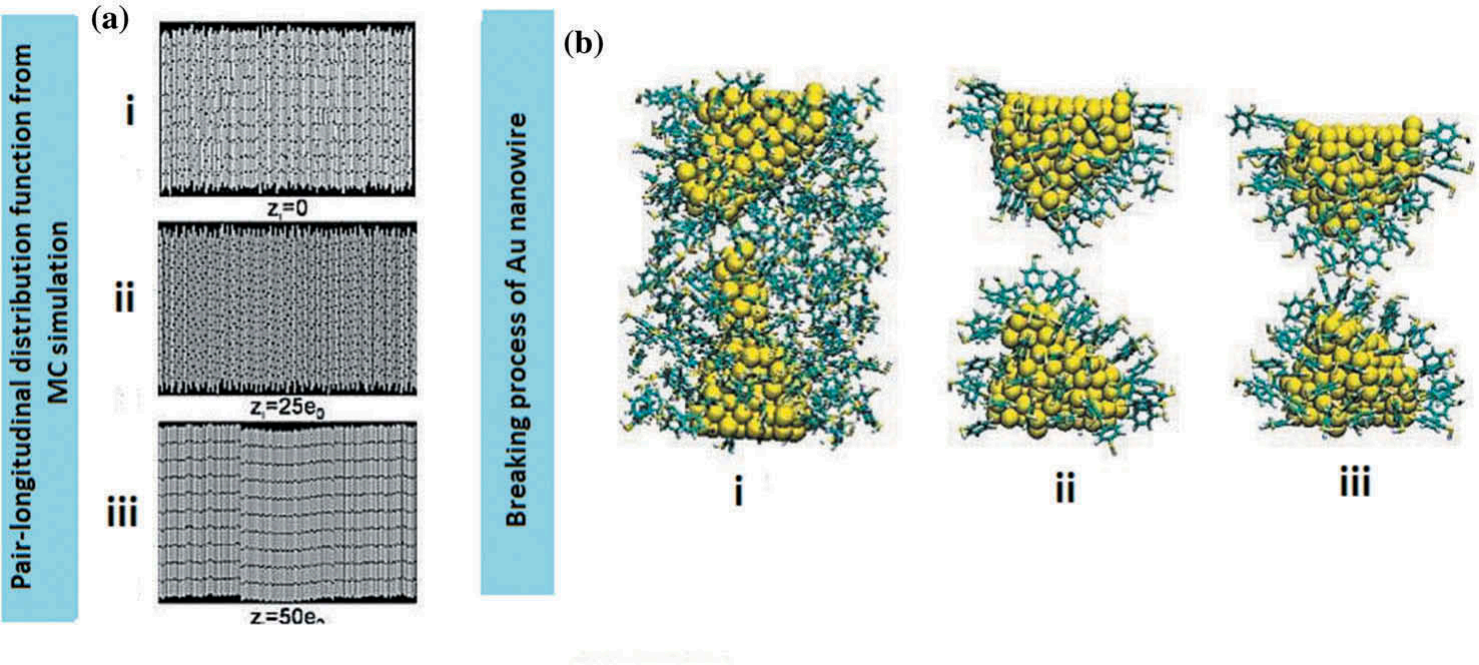
(a) A 4 µm nanowire with various end charge shows the pair-longitudinal distribution function from MC simulation [290]. (b) Snapshot of a 256-atom Au nanowire, which broke in solvent and then reconnected, bridging a Au-BDT-Au junction at room temperature. The order is: i – breaking at Δz = 2.3 nm, ii – reconnecting at Δz = −0.5 nm and iii – reconnecting at Δz = −0.1 nm [291]. (*Adapted with permission from* [290] *copyright (2010) American Chemical Society, reprinted with permission from* [291] *Copyright (2010) American Chemical Society).*

The random displacement points in the *x* and *y* directions give effect in the movement which results in a uniformly distributed surface displacement. The maximum value of the surface displacement can reach 0.1*d*, where *d* stands for diameter. Generally, the surface displacement involved the total Columbic potential *U_total_* and hence, the configuration *ΔU* obtained with certain probability measured as considered in the work by Triplett et al. [290]. Each of the simulations is equilibrated for a minimum MC step, which is the average of one attempted movement per nanowire. At the atomic level, a minimum of 10 production cycles is set for each nanowire length with the output of one production cycle is taken as an input to the next one. The snapshots presented in Figure 11(a) indicates the simulation of 4 µm Au nanowire for no end charges (zeta potential, *z* = 0). As the *z* is increased, there is an increase in smectic order as the well-defined smectic peak layer is present. The broad peak with a sharp central peak indicates the average mass of the wire rows. The MC simulation is capable in finding the lowest end-point charge that further indicated the flexural deformation within any point of atoms along the nanowires.

A MC-embedded MD simulator can be used to investigate the bonding structure and elongation behaviour of metal nanowires [291]. It is desirable to increase the accuracy of the multi-time-scale simulation that provides insights on the structural nature of Ag, Au and Cu nanowires in a sophisticated environment. The concerning situation is the presence of sandwiched molecules in between the metal nanowires contact. The universal force field is used to optimise the structure as rigid molecules using MC simulation. The simulation typically runs under specific designated temperature with specific simulation box lengths in the *x-, y*- and *z*-dimensions with periodic boundary conditions are applied in all direction orientations. For instance, the intrinsic local bonding structure of benzene dithiolate (BDT) and Au nanowires is simulated based on the adsorption of BDT molecules on Au nanowire surface. In the presence of additional molecule between the interfaces of Au nanowires, the elongation behaviour found to be significantly decreased from the simulation result. The breaking of the Au nanowire is on 256-atom at room temperature and that subsequently brought the Au-BDT-Au junction in contact (Figure 11(b)). The breakage of the Au nanowire is only allowed when the solvent is evaporated under the ambient conditions [192]. After removing the non-bonded BDT monolayer, the rapid breaking process takes place in the MC simulation. The simulation results reflect the real situation of stretching Au nanowire joint in the experiment.

Another essential function of MC simulation is when the Morse potential [292] with the function of temperature variation, number of atoms involves and the type of force exerts on the nanowires are included in the inter-atomic interaction analysis. The Morse potential parameters [293] governed the reproducibility of experimental lattice constant, bulk modulus and the cohesive energy of bulk metal nanowires. For example, force relaxation occurs at each of yielding step takes place at smaller strains at 600 K, and the elongation is at 300 K describes the breakage of Au nanowires [294]. The number of atomic layers at the top and bottom remains although the wires are elongated. The atomic arrangement after the yield undergoes abrupt slipping typically along the {111} plane for Au nanowires with the numbers of atomic layers increases from 5 to 6 and further build up to the fourth with the number of atomic layers becomes 8. After the breaking, the wire shrinks quickly, and the necking occurs as to release accumulated elastic energy. The simulation of shear deformation responsible for the slip happens on the plane perpendicular to the wire axis. The first yield takes place just after the disordered arrangement. The shear deformation proceeds in alternating elastic and yielding stages where the number of atomic layers decreases from 5 to 4 at the first yield. This suggests that the invariant in the elastic constant depends upon the size of nanowires.

### 4.4. Summary

The distinctive mechanical properties of metallic nanowires arise from dislocation-twin boundaries interactions which differ from the dislocations-grain boundaries and dislocation-dislocation interactions. Hence, the twin boundaries with perfect structures are crucial for strengthening metallic nanowires. Their characteristics deformation modes (e.g. ductile and brittle) are affected by the wire diameter and temperature. The reported simulations used realistic conditions to mimic the reactions and mechanisms of failure (brittle-to-ductile) in nanowires, and the effects of nanowire diameter and temperature were observed.

The computational methods, especially the atomistic MD simulation, are beneficial when dealing with the investigation of the structural assembly and thermodynamic properties of Ag, Au and Cu nanowires. Nevertheless, the values obtained are considerably overestimated, as the method does not take into account the sink-in or the barrelling effects of these nanowires. These effects, including the specific properties of different types of nanowires (crystallinity) and the effects of lateral confinement lack, all take into account in the FEM simulations. The MC modelling and its consideration of field-effect and other similar simulations are more complicated than the conventional ones due to the presence of additional boundary layer. In the best case, the influences of the conductive charges are taken into account and usually achieved by solving the MC simulation. These considerations indicate that physical modelling and simulation gives rise to several effects including field effect and charge transport that then translated to solve the multiscale problem inherent in the real products. Hence, quantitative understanding and predictive simulations allow for better rational design and the optimisation of the nanowire products. A summary of representative simulations of the mechanical properties of Ag, Au and Cu nanowires is presented in Table 3.

## 5. Conclusions and future trends

As Cu is much cheaper and more abundant than Ag and Au, 1D Cu nanostructures are important for a broad range of science and technology fields. In spite of recent advances in their production, challenges and opportunities remain, especially those related to the reaction mechanisms and improving the synthesis efficiency.

High precision micromechanical testing devices have been utilised in intriguing quantitative mechanical tests of nanowires systems. With details for improvement in the design and laboratory set-up, these nanowires can be tested to ensure the accuracy and validity of the measurements. Micromechanical tests efficiently facilitate the probing of desired structural-mechanical property at the nanoscale, with the possibility of testing a wide variety of different small-dimensional nanoscale materials. Most of the techniques used in this field are frequently employed without much variation regarding the equipment setting and parameters, however, for these nanowires, recent trends have been towards improving the compatibility of the system with the techniques. Despite these advancements, new micromechanical testing methods need to be developed. Also, the limitations exist within these techniques, and the harsh natures of the necessary conditions of metal nanowires need to be addressed.

Ag, Au and Cu nanowires are used in numerous applications. However, the understanding of their mechanical properties remains elusive. Most of the simulations reveal that size-dependent nanomechanical properties of nanowires for diameter below 10 nm are influenced by the nanowire structure, surface stress and defect formation mechanism. Free surfaces are critical in determining the mechanical behaviour of nanowires as they influence intrinsic deformation (compressive or tensile) and dislocation. The quantitative and qualitative agreements between experimental measurements and modelling predictions highlight the utility of atomistic tools in examining the mechanical behaviour of metal nanowires.

Regarding the nanomechanical characterisation, most researchers find their own solutions from the integrated technologies based on the stress–strain relationships, atom-by-atom structural and chemical analysis as well as the electronic properties to suit the emerging applications. Overall:

- For polycrystalline metallic nanowires, atomic-scale imaging is compulsory. Dynamic atomic-scale imaging allows monitoring and controlling strain and stress for nanotechnology applications.
- Microscopic deformation mechanisms, which involve grain boundary sliding, rotation and diffusion, are crucial for developing novel nanowire materials.
- Progress in this field requires atomic-scale understanding of the twinning nucleation and propagation processes in single-crystalline and poly-crystalline nanowires.

There are still many challenges in this field which are crucial for applications, such as increasing the yield of metallic nanowires, optimising their defect density and improving their mechanical properties.
